# A homology-based 3D model and structure–function studies reveal key elements for divalent metal ion transporter ZIP8 (*SLC39A8*) function

**DOI:** 10.1016/j.jbc.2025.110930

**Published:** 2025-11-11

**Authors:** Sven P. Baumann, Gergely Gyimesi, Giuseppe Albano, Daniel G. Fuster, Matthias A. Hediger, Jonai Pujol-Giménez

**Affiliations:** 1 Department of Nephrology and Hypertension, Inselspital, University of Bern, Bern, Switzerland; 2 Department of Biomedical Research, Inselspital, University of Bern, Bern, Switzerland; 3 Graduate School for Cellular and Biomedical Sciences, University of Bern, Bern, Switzerland

**Keywords:** membrane transport, ZIP8, SLC39A8, solute carrier protein, SLC, manganese, zinc, iron, metal ion homeostasis, bicarbonate, cotransport, homology-based 3D model, structure–function studies, mutagenesis

## Abstract

The divalent metal transporter ZIP8 (Zrt/Irt-like protein 8; *SLC39A8*) plays a pivotal role in maintaining the homeostasis of essential micronutrients, such as manganese (Mn^2+^), zinc (Zn^2+^), and iron (Fe^2+^). Genetic variants of *SLC39A8* have been associated with a variety of human diseases, including neuropsychiatric disorders, Crohn's disease, and obesity. To gain insight into ZIP8-mediated metal transport, we generated a homology-based 3D model and identified the amino acid residues constituting metal-binding sites 1 (M1) and 2 (M2). Mutagenesis of residues N315, E344, and D318, which form M2, resulted in a complete loss of function, suggesting that M2 plays a central role in the binuclear metal center of ZIP8. Conversely, mutagenesis of residues H314, E343, and D410, which form M1, retained functional activity but with significant alterations: Residue H314 was found to affect substrate selectivity, whereas residues E344 and D410 were identified as essential for the transport of Fe^2+^ and Mn^2+^. Furthermore, residue H347 was found to influence the metal transport turnover rates. These findings indicate that M1 provides accessory functions to ZIP8 activity, including maximal transport rates and/or enhanced substrate selectivity. Furthermore, the present study provides the first direct evidence for Zn^2+^–HCO_3_^-^ cotransport by human ZIP8 and provides insights into HCO_3_^-^ modulation of metal transport. In addition, we have identified a novel metal-binding site, termed M4, formed by residues D311, E348, and D351. Overall, the present study reveals new insights into the structure and metal transport function of ZIP8 and provides a new framework for interpreting functional defects and designing potential therapeutic interventions.

Many biological processes involve the action of transition metal ions. Five of the first-row transition metals of the periodic table are essential, and four of them, manganese (Mn), iron (Fe), cobalt (Co), and copper (Cu), are redox active (1). The latter are found in the catalytic sites of metalloenzymes, enzymes that catalyze vital reactions in biosynthesis and metabolism, and where metal ions act mainly as Lewis acids or as redox-active sites (2). The fifth essential transition metal is zinc (Zn^2+^), which is the only redox-inactive first-row essential transition metal ion, exerts its function by playing a structural role in metalloenzymes that catalyze hydrolytic and other nonredox reactions (1). Zn^2+^ also plays a critical role in stabilizing the structure of transcription factors, including Zn finger proteins (3). Almost 40% of all enzymes are metal ion dependent (4).

Although required for a wide variety of biological processes, essential transition metal ions in excess can be toxic for cells, especially through the formation of reactive oxygen species (5, 6, 7, 8, 9). Conversely, deficiency of divalent metal ions has been linked to a wide variety of human diseases and pathological conditions, such as anemia, hemochromatosis, diabetes, immune disorders, cancer, or neurological disorders and lack of host defense against pathogens (8, 9, 10, 11, 12, 13, 14, 15, 16, 17). The transport of divalent transition metal ions, such as Mn^2+^, Fe^2+^, and Zn^2+^, across cellular membranes is therefore thoroughly regulated by specific solute carriers (SLCs). Among the different divalent metal ion membrane transporters, the SLC39 (solute carrier 39) family, alternatively called Zrt/Irt-like protein (ZIP) family, is well known for its critical role in maintaining appropriate cellular levels of Zn^2+^.

Moreover, two members of the ZIP family, ZIP8 (*SLC39A8*) and ZIP14 (*SLC39A14*), have been shown to play important roles in maintaining Mn^2+^ homeostasis (18, 19). Mn^2+^ is an essential component of various enzymes crucial for living cells, including mitochondrial superoxide dismutase, pyruvate carboxylase, arginase, and Golgi-resident glycosylation enzymes (20, 21). Loss-of-function mutations of *SLC39A8* have been shown to cause systemic Mn^2+^ deficiency (22, 23) because of impaired ZIP8 protein expression (13, 24, 25). In addition, genome-wide association study and other studies have shown that the genetic variant of *SLC39A8*, rs13107325 (A391T), is associated with numerous pathological conditions, such as schizophrenia, Crohn's disease, scoliosis, and obesity, many of which are reported to be the result of defective Mn^2+^ homeostasis (18, 23, 26, 27, 28, 29, 30, 31, 32). In contrast, loss-of-function mutations in *SLC39A14* result in Mn^2+^ overload, primarily affecting the central nervous system because of Mn^2+^ toxicity, leading to dystonia–parkinsonism (33, 34, 35, 36, 37). In addition to Mn^2+^, ZIP8 and ZIP14 also play an important role as transporters of Zn^2+^. ZIP8 was first discovered as a Zn^2+^ transporter after being induced in monocytes by microbial challenge, leading to intracellular Zn^2+^ accumulation (38). ZIP8 regulates the innate immune system through NF-κB activity in macrophages and monocytes, influencing immune function during mycobacterial infection and inflammation. ZIP8 has also been reported to be expressed in human T-cell lysosomes, releasing stored Zn^2+^ into the cytosol to inhibit calcineurin and thereby increase interferon-γ expression (16). Moreover, the *SLC39A8* A391T (rs13107325) variant has been reported to result in increased innate immune signaling, contributing to the pathogenesis of schizophrenia (39). Regarding ZIP14-mediated Zn^2+^ transport, its expression has been shown to be induced in response to proinflammatory stimuli, resulting in a decrease in serum Zn levels (40). ZIP8 and ZIP14 have also been reported to contribute to the clearance of non–transferrin-bound Fe^2+^ from the circulation under Fe overload conditions (41, 42).

In addition, recent studies have revealed the existence of potential Cu^2+^ transporters among ZIP family members, such as ZIP5 (*SLC39A5*) and ZIP10 (*SLC39A10*), whose contributions to human physiology remain to be elucidated (43).

Different transport modes have been proposed for members of the ZIP family, including cotransport of Zn^2+^ and HCO_3_^-^ (44, 45) or Zn^2+^ and H^+^ (46), channel-like transport (47) and/or pH- and voltage-modulated uniport (48, 49). However, for most ZIPs, the transport mechanism is not fully described or remains controversial. In terms of the structural properties of ZIPs, the available information is limited to data on their ortholog from *Bordetella bronchiseptica* (BbZIP) (50, 51) and an isolated soluble domain from human ZIP4 (*SLC39A4*) (52). In addition, several computational 3D structural models have been generated in recent years, including the Rosetta *ab initio* prediction model of human ZIP4 (53) or the homology-based models of human ZIP2 (*SLC39A2*) generated by our group (49). According to the available structural information, the ZIP fold is described as a transmembrane domain composed of eight pseudosymmetrically arranged transmembrane helices (TMHs) with both the N- and C-terminal regions of the polypeptide chain exposed to the extracellular side. The eight TMHs form two separate helix bundles consisting of TMH1, TMH4, TMH5, and TMH6 and TMH2, TMH3, TMH7, and TMH8, respectively (54). Between the two bundles, there is a central ion-binding region with the metal-coordinating residues located in TMH4 and TMH5 (54). According to the available crystal structures of BbZIP, the ion-binding region is formed by a binuclear metal center, which is proposed to have asymmetric functions (50). The metal coordination sites of the binuclear center are designated M1 and M2. Subsequent studies with human ZIP4 have shown that M1 is essential for Zn^2+^ transport, whereas M2 is auxiliary, as demonstrated by mutagenesis studies showing that M2 is not required for Zn^2+^ transport or its binding to M1 (51). Consistent with this, human ZIP2 has been shown to harbor a single metal coordination site corresponding to M1, with a charged lysine residue replacing the putative binding site for the second metal ion in M2 (49). Recently, a new cryo-electron microscopy structure of BbZIP revealed the presence of a third metal coordination site, referred to as M3, capable of regulating the functional activity of the transporter. M3 is located in the cytoplasmic exit pathway, and when intracellular Zn^2+^ levels are elevated, cytoplasmic Zn^2+^ ions interact with two histidine residues located in an adjacent extracellular loop to maintain the transporter in a locked inward–open conformation (55). The study also showed that the transporter forms a homodimer, with each protomer containing nine TMHs and three metal ions. In addition, another recent crystallization study with BbZIP showed that ZIP proteins function as two-domain elevator-type transporters (54).

In the present work, we focused on human ZIP8, a key transporter of both Zn^2+^ and Mn^2+^, whose genetic variants have been associated with a variety of diseases as mentioned above. ZIP8 is widely expressed in mammalian tissues, where it is commonly found as an N-glycosylated protein on the cell surface (13), although it has also been reported to be expressed in the membranes of intracellular organelles, such as lysosomes (16) and mitochondria (56). In the case of polarized cells, such as intestinal (ileum), kidney proximal tubule and lung epithelial cells, it is predominantly found on the apical side (56, 57, 58). Regarding its natural substrates, ZIP8 is a broad-spectrum divalent metal ion transporter that has been shown to be able to transport Mn^2+^, Fe^2+^, Cd^2+^, Co^2+^, and Zn^2+^ (41, 59). Interestingly, at a specific site in TMH5, which is part of the M1 site, ZIP8 has a glutamic acid (E343) residue instead of the histidine residue conserved in most other ZIPs. This variation found in ZIP8 and ZIP14 has been proposed to be the key to their expanded metal selectivity (60).

Metal ion transport by ZIP8 and its closely related paralog ZIP14 has been reported to be electroneutral, suggesting that the transport activity requires the symport or antiport of additional ionic species (41, 44, 45, 61, 62). The functional activity of both transporters is stimulated by extracellular bicarbonate (HCO_3_^-^), and accordingly, a Zn^2+^–HCO_3_^-^ cotransport mechanism has been proposed (45, 61). Another study suggested that ZIP8 mediates the transport of an electroneutral complex containing three ions: Zn^2+^, HCO_3_^-^, and selenite (HSeO_3_^-^) (63). However, there is no experimental evidence for the cotransport of HCO_3_^-^ and/or HSeO_3_^-^ together with Zn^2+^ or Mn^2+^. In addition, the lack of a crystal structure or validated computational model for ZIP8 has further hindered the confirmation of the proposed cotransport mechanism and the understanding of its physiological function.

To address these knowledge gaps, a homology-based 3D structural model of human ZIP8 was generated using the inward–open BbZIP crystal structure as a template (50). Subsequently, *in vitro* experiments were conducted to identify structural elements that are key to ZIP8 functional activity. In this regard, our experiments allowed the identification of the residues that constitute the metal coordination sites and the analysis of their individual contributions to different transport properties, including kinetics and substrate selectivity. Moreover, we present the first direct evidence for Zn^2+^–HCO_3_^-^ cotransport, and in addition, provide insights into the HCO_3_^-^ modulation of the metal transport process.

## Results

### Structural model of ZIP8 based on the BbZIP crystal structure and single-point mutagenesis

**Figure 1:**
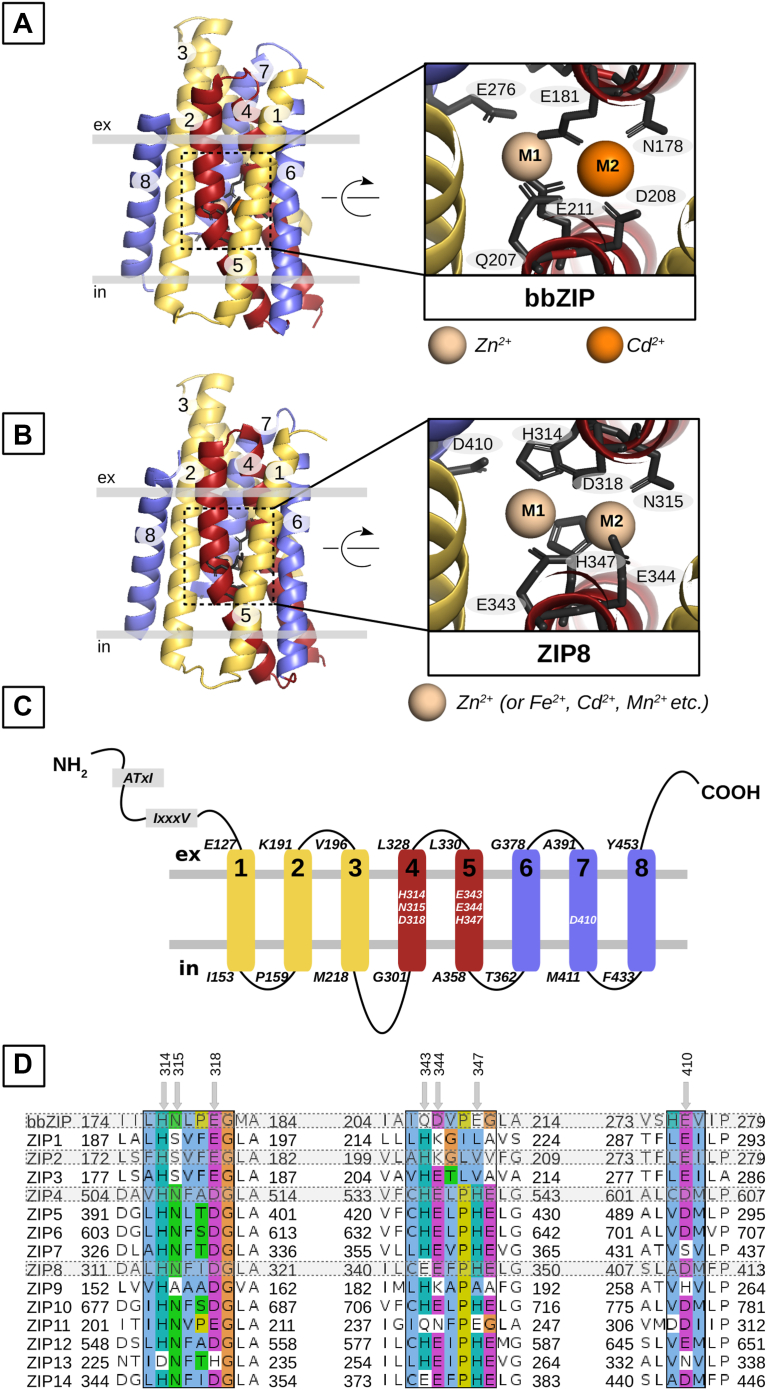
**Homology-based structural model of human ZIP8**. *A,* side view of the crystal structure of BbZIP and close-up view of the metal-binding site. The highlighted residues comprise the metal-binding site. *B,* side view of the ZIP8 structural model and close-up view of the metal-binding site. The highlighted residues are homologous to those predicted to form the BbZIP-binding site. The ZIP8 model was generated in the presence of Zn^2+^ at the M1 and M2 metal ion–binding sites. Additional metal ions are shown in parentheses, which bind to M1 or M2 in different ways based on metal transport experiments shown later in this study. *C,* topological model of ZIP8 showing amino acid positions (*black*) of each transmembrane helix (TMH) and predicted binding site residues (*white*). For *A*–*C,* the TMHs are numbered 1 to 8 and colored according to their 3 + 2 + 3 structural arrangement: TMH 1 to 3 (*yellow*), TMH 4 to 5 (*red*), and TMH 6 to 8 (*blue*). The membrane bilayer is represented by *light gray lines*, which delimit the intracellular (in) and extracellular (ex) spaces. *D,* a multiple alignment of the human SLC39/ZIP family and the prokaryotic homolog BbZIP is presented. Residues predicted to participate in metal coordination are indicated by an *arrow*. Conserved residues are colored according to the properties of their side chains (Jalview, Clustal color scheme). BbZIP, Bordetella bronchiseptica ZIP; SLC39, solute carrier 39; ZIP8, Zrt/Irt-like protein; Zn, zinc.

To investigate the metal ion–binding site of ZIP8, we generated a 3D structural model of human ZIP8 based on the inward–open BbZIP crystal structure available in the Research Collaboratory for Structural Bioinformatics Protein Data Bank (PDB ID: 5TSA) (52) and MODELLER software (University of California) (64). Figure 1*A* shows the side view of the structural model from the crystal structure of BbZIP, and Figure 1*B* shows the side view of our new homology model for ZIP8. Figure 1*C* shows a topological version of the ZIP8 model. Both models show eight TMHs and a large intracellular loop between helices 3 and 4, typical of all SLC39 family members. The structure and function of this loop is not yet fully understood, and because of the absence of structural information from the bacterial structure, we did not model this region.

Consistent with the bacterial structure, the ZIP8 model is inwardly open and shows a 3 + 2 + 3 architecture, that is, it consists of two 3-TMH bundles in opposite orientations connected by two linker TMHs (50) (Fig. 1, *A*–*C*). Our ZIP8 homology model is consistent with the AlphaFold model of ZIP8 (UniProt accession: Q9C0K1) in terms of overall structural arrangement, except that TMH3 crosses in front of TMH7 in our model, whereas it is located at the edge of ZIP8 next to TMH8 in the AlphaFold model.

For the BbZIP and ZIP8 models shown in Figure 1, *A* and *B*, a close-up view of the metal-binding site is shown on the *right panels*. To identify functionally interesting residues in this binding site, multiple protein sequence alignments were performed on all human SLC39 family members and the bacterial ZIP (bbZIP) (Fig. 1*D*). They show that bbZIP and ZIP8 share a conserved metal ion–binding site, with bbZIP having only one histidine (H177, corresponding to H314 in ZIP8), whereas human ZIP8 has a second histidine residue in the metal-binding site, H347. Interestingly, most human ZIPs have a third histidine residue corresponding to the position where ZIP8 has a glutamate residue (E343). Residue E343 has been proposed to be responsible for the expanded metal selectivity of ZIP8 (65, 66). We also found that the three residues of the ZIP8-binding site, N315, D318, and E344, are conserved in almost all ZIP proteins. Based on the structural analogy with BbZIP (Fig. 1*A*), we identified the most likely metal coordination sites within our newly generated human ZIP8 structural model (Fig. 1, *B* and *C*).

According to our ZIP8 structural model (Fig. 1*B*), a binuclear substrate-binding site is plausible, consisting of metal coordination site 1 (M1), comprising of amino acid residues H314 (TMH4), E343 (TMH5), H347 (TMH5), and possibly D410 (TMH7), and metal coordination site 2 (M2), comprising of amino acid residues N315 (TMH4) and E344 (TMH5), and the bridging residue D318 (TMH4), which can interact with both M1 and M2. In addition, we hypothesize that residue D410 (TMH7), just opposite M1, could potentially be involved in the binding of divalent metal ions. In contrast to M2, which is more buried in the membrane-spanning region of the transporter structure, M1 faces a solvent-accessible vestibule and can potentially accommodate larger ions.

To validate the contribution of each of the mentioned residues (H314, E343, H347, N315, E344, D318, and D410) to metal coordination, we generated a series of single-point variants for each of these amino acid residues and experimentally characterized the effect of these mutations on the basic transport properties of human ZIP8, that is, transport kinetics and substrate selectivity. For this purpose, H residues were mutated to A or N residues; E residues were mutated to A, Q, K, D, or R residues; and D residues were mutated to A, E, or N residues. The goal of the mutations was to alter the electrostatic equilibrium of the proposed binuclear center by (1) removing charged side chains (A mutants), (2) replacing charged residues with polar residues (Q and N mutants), or (3) reversing the charge (K or R mutants) in order to assess the contribution of each residue to metal binding and transport kinetics. In addition, mutations that remove larger side chains, such as H to A, could potentially alter certain structural constraints that are key to substrate selectivity or rate-limiting steps of the transport cycle.

It is worth noting that ZIP8 has two sequence motifs (DxxxT and ATxI) that have been found in HCO_3_^-^ transporters and are associated with HCO_3_^-^ binding in the SLC4 family (67). However, while these sequences are located in TMHs 8 and 10 in SLC4 family members, they are located in the unstructured extracellular N terminus (residues 1–127) in ZIP8 (Fig. 1*C*). Due to the lack of structural information, we did not focus on this region but rather on the metal ion–binding site of ZIP8.

### Plasma membrane expression and functional activity of ZIP8 single-point variants

**Figure 2:**
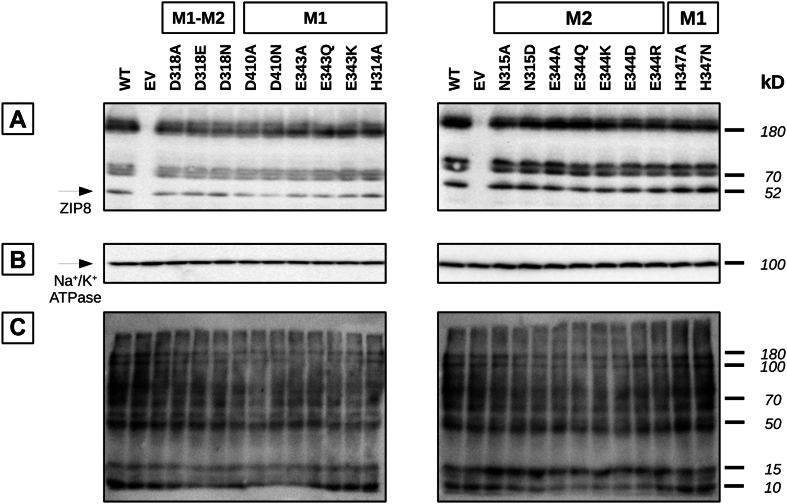
**Plasma membrane expression of the generated human ZIP8 variants**. The cell membrane surface of HEK293T cells overexpressing the indicated DNA constructs was labeled with sulfo-NHS-LC biotin. Subsequently, the labeled membrane proteins were isolated using streptavidin–agarose beads, and their expression levels were determined by Western blot analysis. *A*, an anti-ZIP8 antibody (≈52 kDa) was used to monitor alterations in the plasma membrane expression of the generated ZIP8 variants. A comparison of the expression levels of WT ZIP8 with those of the indicated variants revealed no statistically significant differences (n = 3). *B*, the Na^+^–K^+^ ATPase α1 subunit (≈100 kDa) was used as control for the membrane protein isolation procedure. *C*, the HRP–avidin antibody was used as an equal loading control. HEK293T, human embryonic kidney 293T cell line; HRP, horseradish peroxidase; ZIP8, Zrt/Irt-like protein 8.

Expression of the generated human ZIP8 mutants at the plasma membrane was determined by Western blotting of samples isolated by cell surface biotinylation (Fig. 2). All the mutants were successfully expressed at the plasma membrane with expression levels similar to those observed for WT ZIP8 (Fig. 2*A*). In addition to the protein-specific band at 52 kDa, additional bands are visible at approximately 70 kDa and 180 kDa. The 70 kDa bands likely correspond to glycosylated and/or phosphorylated forms of ZIP8 (68), whereas the 180 kDa bands may represent heat-induced protein aggregates that formed during sample boiling. Na^+^/K^+^-ATPase expression was assessed in the same samples as a positive control for cell surface isolation (Fig. 2*B*), whereas biotin was assessed as a sample loading control (Fig. 2*C*).

**Figure 3:**
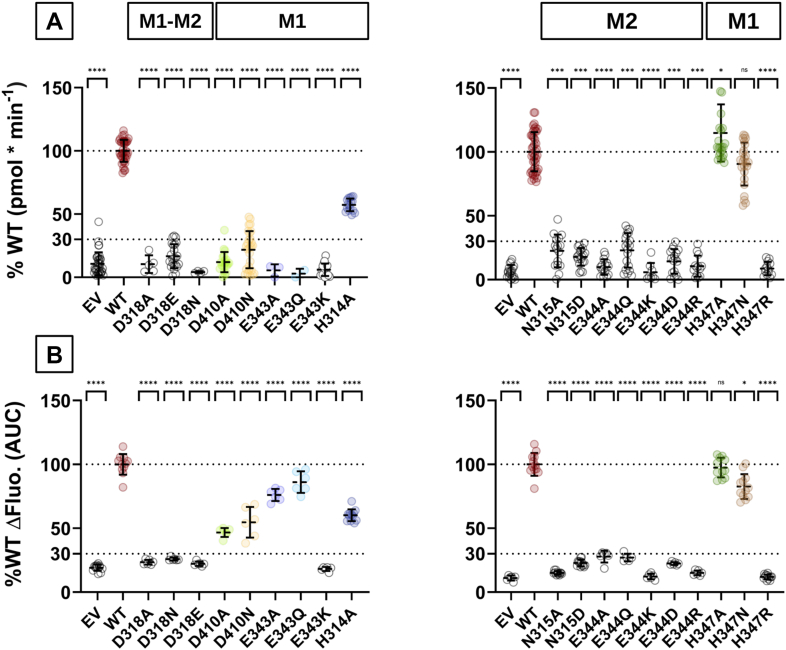
**Functional activity of the generated human ZIP8 variants**. HEK293T cells were transiently transfected with the empty vector (EV), WT human ZIP8, or the indicated ZIP8 variants and subsequently used to measure metal ion transport. *A*, the uptake of radiolabeled iron (^55^Fe^2+^) was determined in ^55^Fe-uptake buffer (pH 7.4) containing 5 μM Fe^2+^. *B*, the uptake of Cd^2+^ was measured in NCF buffer (pH 7.4) containing 2 μM Cd^2+^. The intracellular accumulation of Cd^2+^ was quantified as the fluorescence signal of the FLIPR Calcium-5 dye. Transport was measured after 15 min of incubation at room temperature. The nonspecific activity was determined in the nontransfected cells and subtracted from the values determined with the transfected cells. The data were normalized to the average functional activity of WT ZIP8 determined in each individual experiment. Data obtained from four independent experiments (N = 8–32) are presented with the corresponding mean ± SD values. The *dashed lines* indicate the thresholds used to categorize the functional activity of the indicated ZIP8 variants as either unaltered (100%) or inactive (less than 30%). Statistical differences between groups were assessed using the one-way ANOVA test, followed by the *post hoc* Tukey's test. WT ZIP8 was compared with either EV or each of the indicated ZIP8 variants. Significance thresholds were defined as follows: *p* > 0.05 (not significant, ns), *p* ≤ 0.05 (∗), *p* ≤ 0.01 (∗∗), *p* ≤ 0.001 (∗∗∗), and *p* ≤ 0.0001 (∗∗∗∗). FLIPR, Fluorescent Imaging Plate Reader; HEK293T, human embryonic kidney 293T cell line; N, number of replicates per group; ZIP8, Zrt/Irt-like protein 8.

The functional activity of the generated human ZIP8 variants was evaluated using the radioisotope ^55^Fe^2+^ transport assay (Fig. 3*A*). All but the H314A, H347A, and H347N variants showed transport activity below the threshold of 30% of that of WT ZIP8, which we considered inactive. The H314A variant reduced the Fe^2+^-transporting activity by 50%, whereas the H347A and H347N variants did not significantly change the activity. Subsequently, to ascertain whether the observed loss of function among the ZIP8 variants was related to the type of metal substrate, we evaluated the Cd^2+^ transport capacity using the Fluorescent Imaging Plate Reader (FLIPR) Calcium-5 fluorescence-based assay (Fig. 3*B*). When compared with WT ZIP8 Cd^2+^ transport, H314A, H347A, and H347N behaved as observed for Fe^2+^ transport, showing a similar reduction in Cd^2+^ transport. In contrast, the D410 and E343 variants, which were inactive for Fe^2+^ transport, showed partial transport activity for Cd^2+^, that is, Cd^2+^ transport was only reduced by 50%, 40%, 20%, and 10% for D410A, D410N, E343A, and E343Q, respectively. Collectively, these results indicate that mutations of residues proposed to be part of the metal coordination site M2, including N315, E344, and the common bridging residue D318, are incompatible with functional activity, suggesting that M2 plays a critical role in metal ion transport. On the other hand, variants of the metal coordination residues in M1, including H314, D410, E343, and H347, show functional activity for Cd^2+^ transport. Remarkably, in the case of Fe^2+^ transport, activity was lost upon the mutation of residues E343 and D410, supporting the notion that these residues affect divalent metal ion substrate specificity.

### Metal ion transport kinetics of the functionally active ZIP8 single-point variants

**Figure 4:**
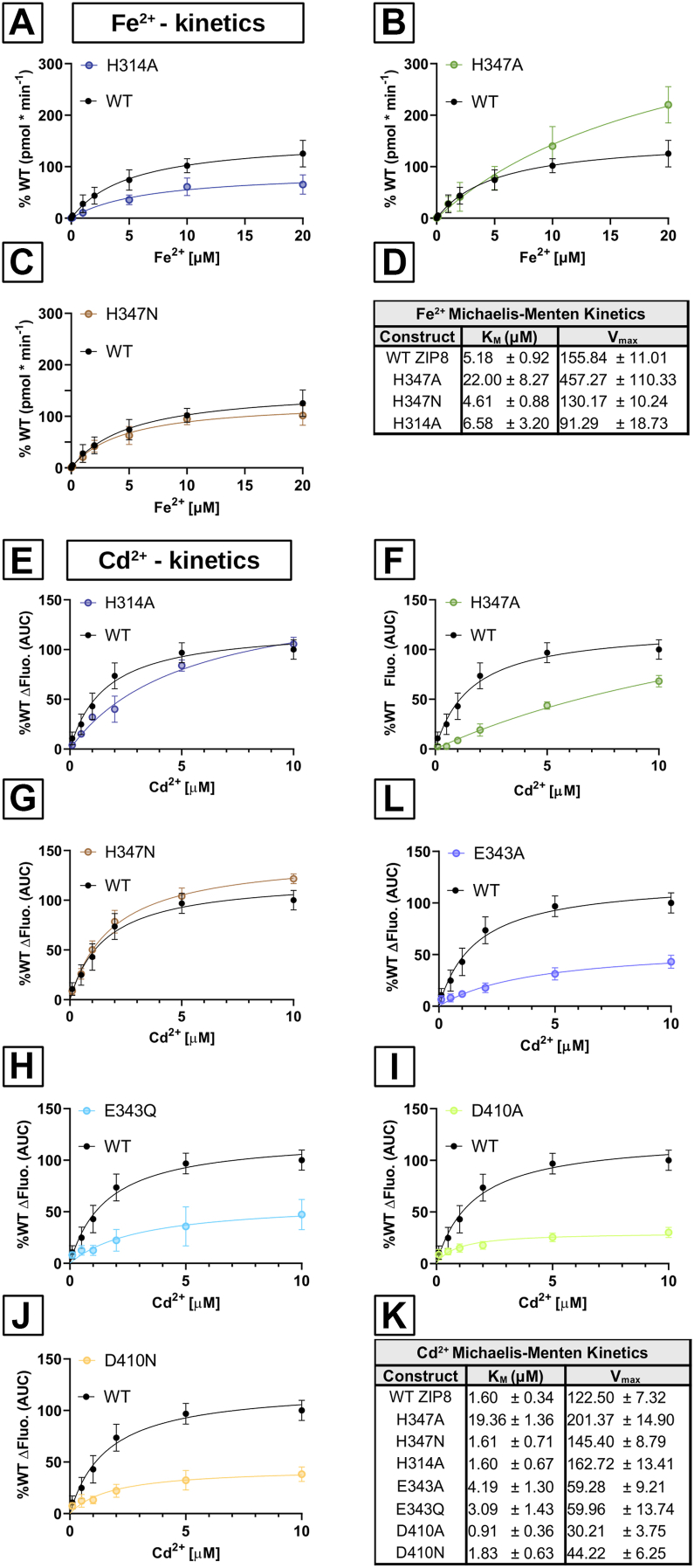
**Divalent metal ion transport kinetics of the active human ZIP8 variants**. Divalent metal ion transport kinetics were determined using HEK293T cells transiently transfected with WT human ZIP8 or the indicated ZIP8 variants. *A*–*C*, the uptake of radiolabeled iron (^55^Fe^2+^) was measured in ^55^Fe-uptake buffer (pH 7.4) containing different concentrations of Fe^2+^ (0.1–20 μM). *D*, the table summarizing the calculated kinetic parameters for Fe^2+^ uptake. *E*–*J*, uptake of Cd^2+^ was measured in NCF buffer (pH 7.4) containing different concentrations of Cd^2+^ (0.1–10 μM). The intracellular accumulation of Cd^2+^ was quantified as the fluorescence signal of the FLIPR Calcium-5 dye. *K*, the table summarizing the calculated kinetic parameters for Cd^2+^ transport. Transport was measured after 15 min incubation at room temperature. The nonspecific activity was determined in the nontransfected cells and subtracted from the values determined with the transfected cells. The data were normalized to the average functional activity of WT ZIP8 determined in each individual experiment. Data obtained from four independent experiments (N = 16–31) are presented with the corresponding mean ± SD values. The *solid-colored lines* represent the fit of the data to the Michaelis–Menten equation. FLIPR, Fluorescent Imaging Plate Reader; HEK293T, human embryonic kidney 293T cell line; ZIP8, Zrt/Irt-like protein 8.

To better understand the role of the proposed metal coordination residues in ion binding and transport by human ZIP8, we used radiolabeled ^55^Fe^2+^ (Fig. 4, *A*–*D*) and fluorescence-based Cd^2+^ transport assays (Fig. 4, *E*–*K*) with cells overexpressing the functionally active human ZIP8 variants (H314A, H347A, H347N, E343A, E343Q, D410A, and D410N) to study the transport kinetics. Compared with WT ZIP8, the H314A and H347N variants showed no significant differences in Fe^2+^ transport kinetics, with both *K*_*M*_ and *V*_max_ parameters in a similar range as WT (Fig. 4*D*). Interestingly, the H347A variant showed a fourfold decrease in affinity for Fe^2+^ with a threefold increase in *V*_max_. This low-affinity/high-capacity effect of H347A indicates an important role of H347 in the metal transport mechanism by ZIP8.

With respect to Cd^2+^ transport, the active variants of residues H347 and H314 exhibited similar effects on Fe^2+^ and Cd^2+^ transport, with comparable effects on their respective metal transport kinetics (Fig. 4, *D* and *K*). Furthermore, the *K*_*M*_ of variants E343A, E343Q, D410A, and D410N were in a similar range to that determined for WT ZIP8 (Fig. 4*K*). However, the calculated *V*_max_ for variants E343 and D410 were twofold and threefold lower than WT, respectively. E343 and D410 are proposed to be part of M1 and, as shown in Figure 3*A*, their variants lost the ability to transport Fe^2+^, whereas for Cd^2+^, they showed a reduced transport rate to about half that of WT ZIP8, without substantial changes in binding affinity. The 50% reduction in *V*_max_ of Cd^2+^ transport may indicate that the E343 and D410 variants exclusively affect the function of M1, whereas M2 might still be functional, reducing the *V*_max_ to half.

### Effect of ZIP8 single-point variants on substrate selectivity

To further evaluate the contribution of the proposed metal ion coordination residues to the substrate selectivity of human ZIP8, we performed metal ion competition experiments challenging Fe^2+^ or Cd^2+^ transport with an excess of transition metal ions, such as Zn^2+^, Mn^2+^, Fe^2+^, Co^2+^, Cu^2+^, or Cd^2+^, as well as the alkaline earth metal barium (Ba^2+^).

**Figure 5:**
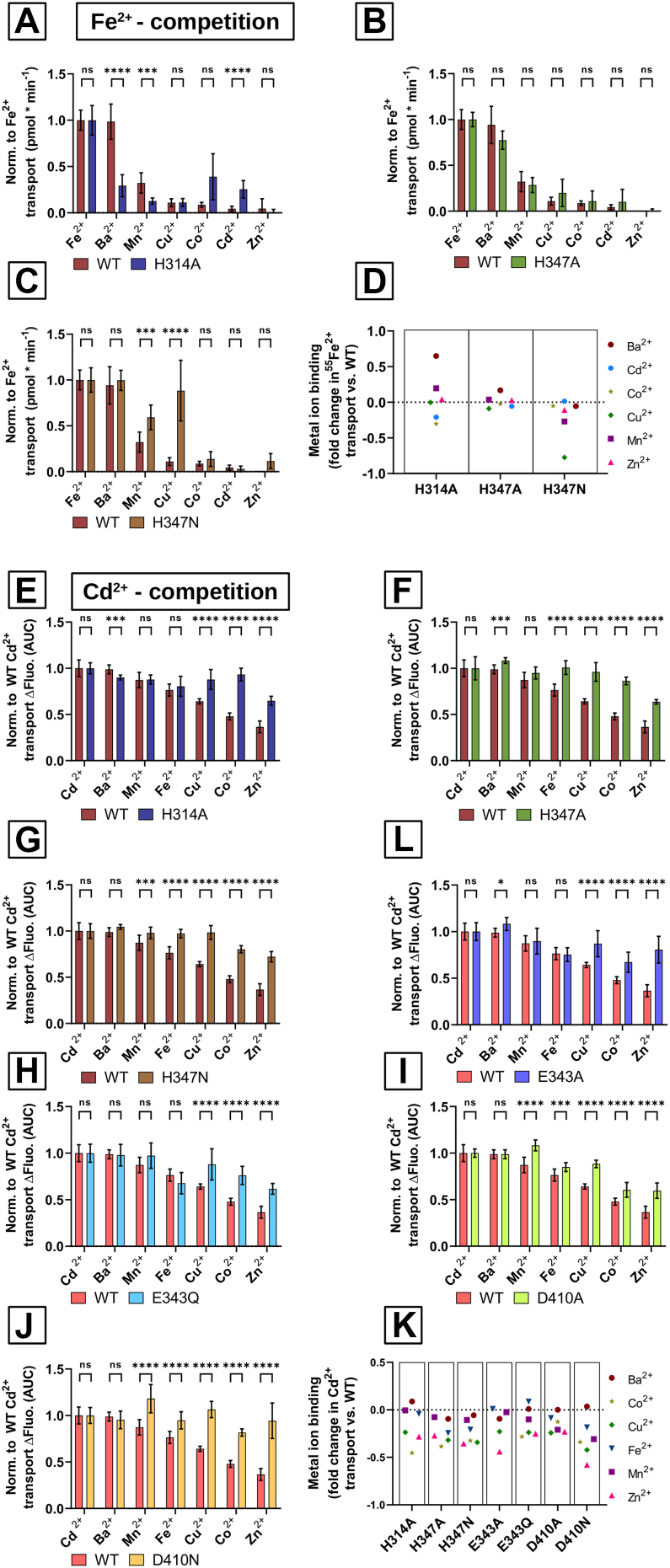
**Divalent metal ion substrate selectivity of the active human ZIP8 variants**. Divalent metal ion (Me^2+^) transport competition experiments were performed using HEK293T cells transiently transfected with the empty vector (EV), WT human ZIP8, or the indicated ZIP8 variants. *A*–*C*, the uptake of radiolabeled iron (^55^Fe^2+^) was measured in ^55^Fe-uptake buffer (pH 7.4) containing 5 μM Fe^2+^ alone (first column) or in combination with 50 μM of the indicated Me^2+^. *D*, the fold change in the inhibition of ^55^Fe-uptake by the indicated Me^2+^ for each of the indicated ZIP8 variants compared with WT control is presented. *E*–*J*, Cd^2+^ uptake was measured in NCF buffer (pH 7.4) containing 2 μM Cd^2+^ alone (first column) or in combination with 20 μM of the indicated Me^2+^. The intracellular accumulation of Cd^2+^ was quantified as the fluorescence signal of the FLIPR Calcium-5 dye. *K*, the fold change in the inhibition of Cd^2+^ uptake by the indicated Me^2+^ for each of the indicated ZIP8 variants compared with WT control is presented. The transport was measured after 15 min incubation at room temperature. The nonspecific activity was determined in the nontransfected cells and subtracted from the values determined with the transfected cells. The data were normalized to the average functional activity of WT ZIP8 determined in each individual experiment. The *y*-axes show the normalized Fe^2+^ (*A*–*C*) and Cd^2+^ (*E*–*J*) transport, where 1 represents the average Fe^2+^ or Cd^2+^ transport of WT ZIP8. ^55^Fe^2+^ uptake was calculated as counts per minute. For Cd^2+^, activity was quantified as AUC (area under the curve). The data obtained from four independent experiments (N = 15–20) are presented with the corresponding mean ± SD values for each of the indicated Me^2+^. Statistical differences between groups were assessed using either the Student’s *t* test or the Mann–Whitney *U* test, depending on the outcome of the sample distribution analysis. WT ZIP8 was compared with each of the indicated ZIP8 variants. Significance thresholds were defined as follows: *p* > 0.05 (not significant, ns), *p* ≤ 0.05 (∗), *p* ≤ 0.01 (∗∗), *p* ≤ 0.001 (∗∗∗), and *p* ≤ 0.0001 (∗∗∗∗). Brackets indicate the groups compared statistically. FLIPR, Fluorescent Imaging Plate Reader; HEK293T, human embryonic kidney 293T cell line; ZIP8, Zrt/Irt-like protein 8.

The substrate selectivity observed for WT ZIP8, determined as inhibition of Fe^2+^ transport (Fig. 5*A*), was Zn^2+^ > Cd^2+^ > Co^2+^ > Cu^2+^ > Mn^2+^. Cu^2+^ is not a verified transport substrate, but several publications (31, 41) have shown that Cu^2+^ competes with known ZIP8 substrates, such as Fe^2+^ and Cd^2+^. Ba^2+^ has been chosen as a negative control because it belongs to group II elements and is therefore not a substrate of ZIP8, as has been demonstrated previously (31). As expected, Ba^2+^ did not inhibit Fe^2+^ transport. Among the ZIP8 variants, the H314A mutant (Fig. 5, *A* and *D*, *left column*) showed not only significant changes in substrate selectivity, most notably the ability to be inhibited by Ba^2+^, but also altered inhibition profiles for Mn^2+^, Co^2+^, and Cd^2+^. The position of H314 on the pathway from the extracellular environment to the proposed M1 and M2 binuclear metal coordination center suggests that H314, located just above the metal-binding site M1 (Fig. 1*B*), may act as a substrate-selectivity filter, blocking the entry of divalent alkaline earth metals such as Ba^2+^. This block may be abolished when H314 is replaced by alanine. In contrast, the H347A variant (Fig. 5, *B* and *D*, *middle column*), which had significant effects on Fe^2+^ and Cd^2+^ transport kinetics (resulting in low-affinity/high-capacity transport; see the tables in Fig. 4, *D* and *K*), does not affect substrate selectivity. However, the H347N variant altered substrate selectivity (Fig. 5, *C* and *D*, *right column*), with a significant reduction in the binding of Mn^2+^ and even more so in the binding of Cu^2+^. This finding suggests that disturbances in the electrostatic equilibrium in the ZIP8-binding site, such as the replacement of the positively charged H with a polar uncharged N at position 347, may also affect the ability of ZIP8 to bind to specific divalent metal ions. The substrate selectivity observed for WT ZIP8, determined as inhibition of Cd^2+^ transport (Fig. 5*E*), was Zn^2+^ > Co^2+^ > Cu^2+^ > Fe^2+^ > Mn^2+^, which is in agreement with that for inhibition profile of Fe^2+^ transport. As expected, Ba^2+^ also did not inhibit Cd^2+^ transport. Moreover, as demonstrated by the transport kinetics studies, Cd^2+^ is a better ZIP8 substrate than Fe^2+^ (Fig. 4*D versus K*). Consequently, the inhibition of ZIP8 transport capacity in the presence of the different divalent metals was overall lower for all the ZIP8 variants. Consistent with the proposed substrate selectivity filter function of H314, variants at this position exhibited significant changes in substrate selectivity when Cd^2+^ was used as a substrate (Fig. 5, *E* and *K*), including the ability of Ba^2+^ to inhibit. In contrast to Fe^2+^ transport, both H347 variants, H347A and H347N, showed altered inhibition profiles (Fig. 5, *F* and *G*). This difference with respect to H347A may reflect different effects on low-affinity/high-capacity transport exhibited by this variant for Fe^2+^ or Cd^2+^ transport (Fig. 4*D versus K*), resulting in different degrees of impaired binding to other divalent metal ions. However, the fact that the H347N variant, showing overall WT-like transport kinetics (Fig. 4), still shows an altered ion inhibition profile implies that H347 affects substrate selectivity, likely by directly binding the transported ion.

Interestingly, neither E343 (Fig. 5, *L* and *H*) nor D410 (Fig. 5, *I* and *J*) variants can transport Fe^2+^ (Fig. 3*A*). Nevertheless, E343 variants still seem to be able to bind Fe^2+^, whereas this ability is almost lost for D410 variants. Likewise, Mn^2+^ binding is unaffected for E343 variants, whereas it is completely lost for all D410 variants. These results confirm that the binding and transport of both Fe^2+^ and Mn^2+^ require the integrity of M1, with residues E343 and D410 being essential for its function. Regarding Co^2+^, Cu^2+^, and Zn^2+^, their binding is significantly reduced for the E343 and D410 variants but not completely abolished. According to the Cd^2+^ transport kinetics determined for the E343 and D410 variants (Fig. 4*B*), the *V*_max_ of Cd^2+^ transport is reduced by more than half, which we interpret as a consequence of M1 being inactive. Therefore, we propose that M2 is also able to bind Co^2+^, Cu^2+^, and Zn^2+^, since M1 is not active for Cd^2+^ transport in these variants. Finally, it is worth mentioning that none of the E343 (except E343A, Fig. 5*L*) and D410 variants showed Ba^2+^ transport, ruling out a possible role as substrate-filtering residues.

### HCO_3_^-^ dependence of the functional activity of ZIP8 WT and single-point variants

**Figure 6:**
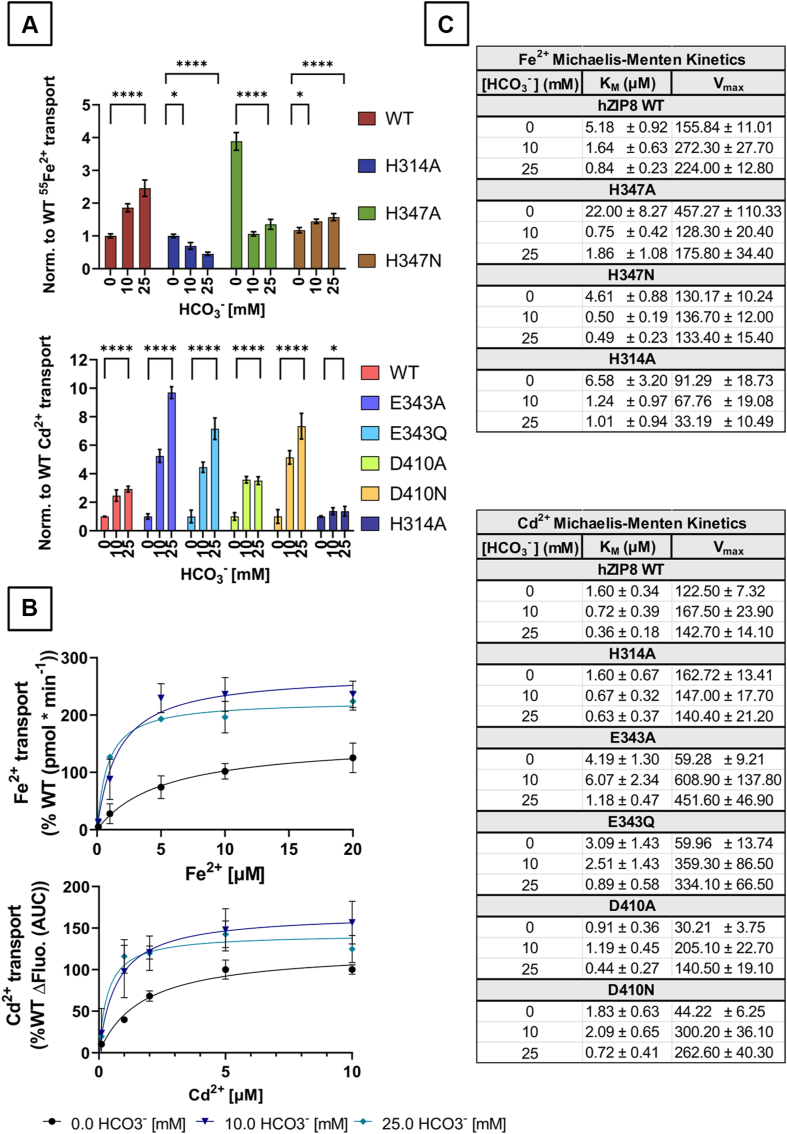
**Effect of extracellular HCO_3_^-^ on divalent metal ion transport kinetics of the active human ZIP8 variants**. Divalent metal ion transport was determined using HEK293T cells transiently transfected with WT human ZIP8 or the indicated ZIP8 variants. All experiments were performed in the absence and presence (10 and 25 mM) of additional NaHCO_3_. *A*, *upper panel*, uptake of ^55^Fe^2+^ (5 μM Fe^2+^). *Lower panel*, uptake of Cd^2+^ (2 μM Cd^2+^). The intracellular accumulation of Cd^2+^ was quantified as the fluorescence signal of the FLIPR Calcium-5 dye. *B*, *upper panel*, uptake of ^55^Fe^2+^ measured in ^55^Fe-uptake buffer (pH 7.4) containing different concentrations of Fe^2+^ (0.1–20 μM). *Bottom panel*, uptake of Cd^2+^ measured in NCF buffer (pH 7.4) containing different concentrations of Cd^2+^ (0.1–10 μM). *C*, the table summarizing the kinetic parameters calculated for each of the ZIP8 variants in the presence of the different extracellular NaHCO_3_ concentrations. Data were normalized to the average functional activity of WT ZIP8 (no added NaHCO_3_) determined in each individual experiment. The *y*-axes show the normalized Fe^2+^ (*A*, *upper panel*) and Cd^2+^ (*A*, *lower panel*) transport, respectively, where 1 represents the average Fe^2+^ or Cd^2+^ transport of WT ZIP8. ^55^Fe^2+^ uptake was calculated as counts per minute. For Cd^2+^, activity was quantified as AUC (area under the curve). The data obtained from four independent experiments (N = 8–11) are presented with the corresponding mean ± SD values for each of the indicated extracellular NaHCO_3_ concentrations. Statistical differences between groups were assessed using the one-way ANOVA test, followed by the *post hoc* Tukey's test. Transport measured in the absence of NaHCO_3_ was compared with the transport measured in the presence of each of the indicated concentrations of NaHCO_3_ for WT and each of the indicated ZIP8 variants. Significance thresholds were defined as follows: *p* > 0.05 (not significant, ns), *p* ≤ 0.05 (∗), *p* ≤ 0.01 (∗∗), *p* ≤ 0.001 (∗∗∗), and *p* ≤ 0.0001 (∗∗∗∗). Variants sharing the same significance level are grouped within the same brackets. FLIPR, Fluorescent Imaging Plate Reader; HEK293T, human embryonic kidney 293T cell line; ZIP8, Zrt/Irt-like protein 8.

Previous works have shown that the functional activity of human ZIP8 is stimulated in the presence of an extracellular favorable electrochemical gradient of [HCO_3_^-^] (44, 45). Therefore, using radiolabeled ^55^Fe^2+^ and fluorescence-based Cd^2+^ transport assays with cells overexpressing human ZIP8 and the generated active variants, we assessed the effect of extracellular HCO_3_^-^ on their functional activity (Fig. 6). It is of note that the zero [HCO_3_^-^] condition is not free of HCO_3_^-^, but it means that no HCO_3_^-^ was added to the extracellular medium. Because there is exogenous HCO_3_^-^ generated from dissolved CO_2_ present in the air, as well as the CO_2_–HCO_3_^-^ generated by the cellular metabolism, there is always a nominal concentration of HCO_3_^-^ in water, which we have estimated to be close to 33 μM HCO_3_^-^ at pH 7.4 according to Henry's law, assuming a pCO_2_ of ∼900 ppm inside the laboratory. Both Fe^2+^ and Cd^2+^ transport by WT ZIP8 was stimulated by twofold to threefold in the presence of extracellular HCO_3_^-^ at 10 to 25 mM (Fig. 6*A*). Among the ZIP8 variants, H314 and H347 mutants seemed to lose the stimulating effect of the extracellular HCO_3_^-^ gradient when using the Fe^2+^ transport assay (Fig. 6*A*, *upper panel*), whereas E343 and D410 variants (Fig. 6*A*, *lower panel*) showed larger stimulation than WT ZIP8 at similar added HCO_3_^-^ concentrations based on experiments using the Cd^2+^ transport assay.

Subsequently, we assessed the transport kinetics of both Fe^2+^ and Cd^2+^ in the presence of different extracellular concentrations of HCO_3_^-^ (Fig. 6, *B* and *C*). Interestingly, for both Fe^2+^ and Cd^2+^ transport by WT ZIP8, the apparent metal ion binding affinity increased along with extracellular [HCO_3_^-^]. Specifically, the affinity for Fe^2+^ increased by threefold to sixfold, whereas the affinity for Cd^2+^ increased by twofold to fourfold upon HCO_3_^-^ addition. Furthermore, for both ions, *V*_max_ reaches the maximum transport rate at 10 mM extracellular HCO_3_^-^, which is 1.4- to 1.7-fold higher for Cd^2+^ and Fe^2+^, respectively, than at zero [HCO_3_^-^] (Fig. 6*C*). Another interesting observation is that at the higher (>10 mM) extracellular [HCO_3_^-^], there is a substantial increase in affinity but a decrease in *V*_max_ (Fig. 6*C*). This finding may suggest that HCO_3_^-^ can help promote the binding of divalent metal ions to the binuclear metal center, which at the same time might make the metal ion release more difficult. Regarding the ZIP8 variants, Fe^2+^ transport kinetics of H347A and H314A mutants showed a much higher affinity to Fe^2+^ in the presence of higher extracellular [HCO_3_^-^], whereas the *V*_max_ is reduced when compared with nominal zero [HCO_3_^-^] condition (Fig. 6*C*, *upper table*). This may indicate that in the absence of the side chains of H347 or H314, the negative charge provided by HCO_3_^-^ may help to restore metal ion binding but not the transport turnover rate, especially in the case of H314 where the increased affinity appears to hinder the release of transported ions, resulting in a decreased *V*_max_. The H347N mutant shows a similar trend, but with almost no effect on *V*_max_, which is independent of extracellular [HCO_3_^-^] for this mutant.

Interestingly, when measuring the effect of extracellular HCO_3_^-^ on Cd^2+^ transport kinetics in the E343A, E343Q, D410A, and D410N variants (Fig. 6*C*, *lower table*), we observed a pronounced increase in *V*_max_, whereas the effect on Cd^2+^ affinity is only apparent at higher extracellular [HCO_3_^-^]. As previously mentioned, these mutants seem to impair the function of M1 (Figs 3 and 4*K*), thus, it can be proposed that in the presence of higher extracellular [HCO_3_^-^], the activity of M1 is recovered. In this regard, given that E343A, E343Q, D410A, and D410N mutations result in the loss of a negative charge in M1, this could potentially be compensated for by HCO_3_^-^, which would provide the lost negative charge and help to coordinate the metal ion, allowing the recovery of the transport function, and finally resulting in an increased *V*_max_.

The effect of HCO_3_^-^ on the functional activity of ZIP8 using a variant that retains the ability to transport both Fe^2+^ and Cd^2+^, such as H314A (Fig. 4, *A* and *E*), was also evaluated. Similar to the findings for Fe^2+^, the H314A mutant showed a much higher affinity for Cd^2+^ in the presence of higher extracellular [HCO_3_^-^] (Fig. 6*C*). Thus, our data indicate that the effect of HCO_3_^-^ in the tested variants is not different among the metal ion substrates tested.

### Effect of the HCO_3_^-^ transport by ZIP8 WT and its single-point variants on intracellular pH

**Figure 7:**
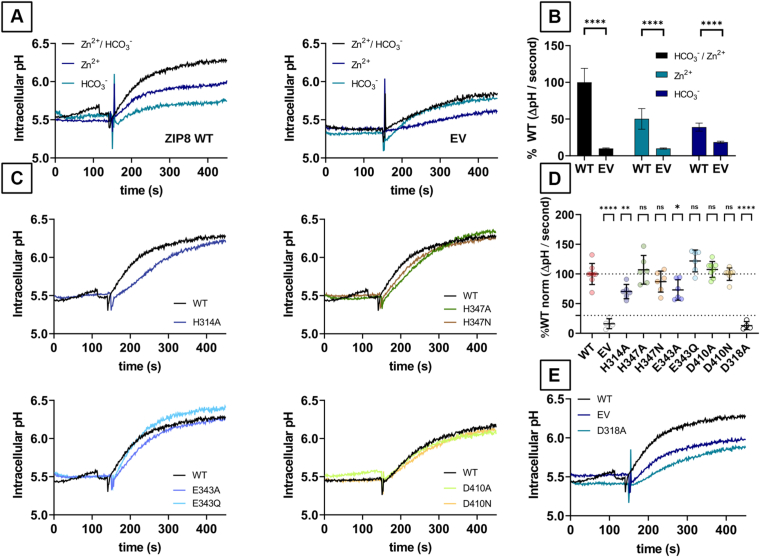
**Effect of divalent metal ion–HCO_3_^-^ cotransport by the active human ZIP8 variants on intracellular pH levels.** Intracellular pH levels were determined using HEK293T cells transiently transfected with empty vector (EV), WT human ZIP8, or the indicated variants. Cells were loaded with the pH-sensitive dye BCECF, and changes in fluorescence intensity were recorded. Fluorescence intensity was converted into pH units using a pH calibration curve. *A*, representative traces showing a comparison of the pH levels calculated with cells overexpressing WT ZIP8 and EV. A baseline was initially established over a period of 150 s. Cells were then injected with 5 μM Zn^2+^ and 25 mM NaHCO_3_, either alone or in combination, and the fluorescence was recorded for an additional 300 s. *B*, average intracellular pH values calculated between 175 s and 275 s from three independent experiments normalized to the reference condition (cells overexpressing ZIP8 WT injected with 5 μM Zn^2+^ and 25 mM NaHCO_3_) are represented as mean ± SD for each of the indicated experimental conditions. *C*, representative traces showing a comparison of the pH levels calculated with cells overexpressing WT ZIP8 and the indicated variants, including a functionally inactive variant (*E*). After the initial baseline of 150 s, cells were injected with 5 μM Zn^2+^ and 25 mM NaHCO_3_ and recorded for 300 s. *D*, average intracellular pH values calculated between seconds 175 and 275 from three independent experiments (N = 4–8) normalized to the reference condition (cells overexpressing ZIP8 WT injected with 5 μM Zn^2+^ and 25 mM NaHCO_3_) are presented as mean ± SD for each of the transfected DNA constructs. Statistical differences between groups were assessed using the Kruskal–Wallis test, followed by *post hoc* Dunn's test. WT ZIP8 was compared with the EV or each the of the indicated ZIP8 variants. Significance thresholds were defined as follows: *p* > 0.05 (not significant, ns), *p* ≤ 0.05 (∗), and *p* ≤ 0.001 (∗∗∗). HEK293T, human embryonic kidney 293T cell line; ZIP8, Zrt/Irt-like protein 8.

As previously stated, extracellular HCO_3_^-^ has been shown to modulate ZIP8 transport function (41). However, to date, there is no direct evidence that HCO_3_^-^ is transported by ZIP8. To address this question, intracellular pH was measured with the pH-sensitive dye BCECF in cells transfected with WT ZIP8 or the empty vector (EV) and perfused with Zn^2+^ and HCO_3_^-^ alone or in combination (Fig. 7*A*). The cells overexpressing ZIP8 showed a highly significant increase in intracellular pH upon perfusion with both Zn^2+^ and HCO_3_^-^ (Fig. 7*B*), compared with those overexpressing the EV. Furthermore, in the zero [HCO_3_^-^] condition (*i*.*e*., only residual [HCO_3_^-^]), the intracellular pH was also significantly increased in the presence of Zn^2+^, whereas in the absence of Zn^2+^, perfusion with extracellular HCO_3_^-^ only slightly altered intracellular pH levels, indicating that HCO_3_^-^ transport *via* ZIP8 is largely dependent on the presence of extracellular Zn^2+^ (Fig. 7*B*). In contrast, EV-transfected cells show no significant intracellular pH changes between the experimental conditions evaluated (Fig. 7, *A* and *B*). These results indicate that the transport of Zn^2+^ and HCO_3_^-^ is specific to the cells overexpressing WT ZIP8 and that HCO_3_^-^-induced changes in intracellular pH are dependent on the presence of extracellular Zn^2+^. In conclusion, our data provide the first direct evidence for HCO_3_^-^ transport by ZIP8 and support the concept of a Zn^2+^–HCO_3_^-^ cotransport mechanism.

Subsequently, intracellular pH was monitored in cells transfected with the indicated ZIP8 variants and perfused with Zn^2+^ and HCO_3_^-^ (Fig. 7, *C* and *D*). Compared with the WT, the H314A and E343A variants showed a 20% to 30% reduction in the ability to raise the intracellular pH, whereas no significant differences were observed for the H347A, H347N, E343Q, D410A, and D410N variants. As expected for the negative controls, cells transfected with EV or the functionally inactive ZIP8 variant D318 did not show a significant increase in intracellular pH throughout the measurements (Fig. 7, *D* and *E*). When compared with the Fe^2+^ transport kinetics measured for the H314A, H347A, and H347N variants in the presence of extracellular [HCO_3_^-^] (Fig. 6*C*), there is consistency between the changes in intracellular pH and the calculated *V*_max_. In the presence of 25 mM [HCO_3_^-^], the *V*_max_ calculated for WT ZIP8 is significantly reduced for the H314 variant, whereas it remains in a similar range for the H347A and H347N variants. This is consistent with the intracellular pH change trend observed with these variants. Conversely, when comparing the Cd^2+^ transport kinetics measured for E343A, E343Q, D410A, and D410N variants in the presence of extracellular [HCO_3_^-^] (Fig. 6*C*), the results are mostly inconsistent. In the presence of 25 mM [HCO_3_^-^], the *V*_max_ calculated for WT ZIP8 is increased by twofold to fourfold in the case of E343A, E343Q, and D410N, or remained in similar range, in the case of D410A. Therefore, it was expected that the variants E343A, E343Q, and D410N would increase the intracellular pH increase rate several fold higher compared with WT. However, no significant differences were observed for these variants compared with WT.

### Assessment of the role of HCO_3_^-^ in ZIP8 function by molecular docking and dynamic simulations

**Figure 8:**
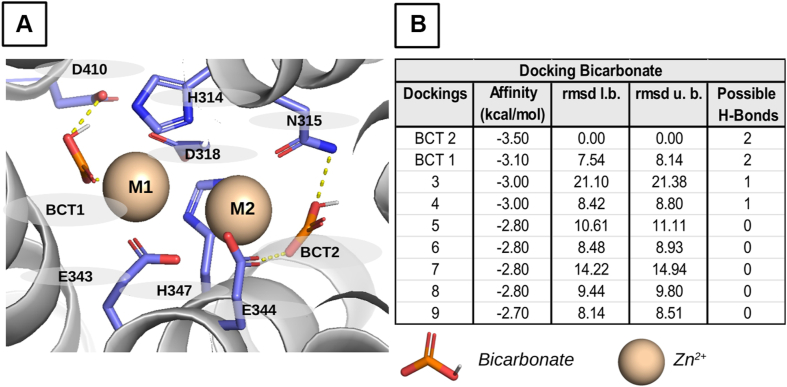
**Evaluation of the potential interactions between HCO_3_^-^ ions and the metal-binding sites of the homology-based model of human ZIP8 by molecular docking.***A*, structural model of the M1 and M2 metal coordination sites of the human ZIP8, bound to Zn^2+^ and HCO_3_^-^ ions, generated on the basis of best scoring conformations identified by molecular docking. The metal ions are represented as *light brown spheres* and potential HCO_3_^-^ (*orange molecule*, BCT1 and BCT2)–ZIP8 chemical interactions are indicated by *dashed yellow lines*. *B*, the table showing the best docking conformations and the corresponding binding free energies. ZIP8, Zrt/Irt-like protein 8.

To further investigate the role of HCO_3_^-^ as a ZIP8 substrate, we used our homology-based model of ZIP8 and molecular docking to identify the potential HCO_3_^-^-binding sites within the metal-binding region (Fig. 8). Molecular docking of HCO_3_^-^ was conducted in the absence and presence of Zn^2+^ metal ions with either the entire protein structure or with a selected area around the binuclear metal center (Fig. 8*A*). Interestingly, in all cases, the binding clusters exhibiting the lowest energies (Fig. 8*B*) were in the same areas close to the M1 and M2 metal coordination sites. The best docking conformation identified for HCO_3_^-^, designated as BCT2, interacts with residues situated in or near the M2 metal coordination site. Specifically, BCT2 is situated between the polar residues of N315 and E344, with which it can potentially form hydrogen bonds. Furthermore, the proximity of the metal ion Zn^2+^ may result in direct interaction with BCT2, thereby providing an additional metal coordination site. In contrast, the second-best docking conformation identified for HCO_3_^-^, designated as BCT1, interacts with residues situated in the M1 metal coordination site. In this conformation, HCO_3_^-^ is located between H347, the negatively charged D410, and the metal ion Zn^2+^. This conformation would allow electrostatic interactions between HCO_3_^-^ and the metal ion Zn^2+^, thereby providing an additional metal coordination site, as well as with residue H347, which according to our experiments seems to be relevant for the metal transport mechanism.

**Figure 9:**
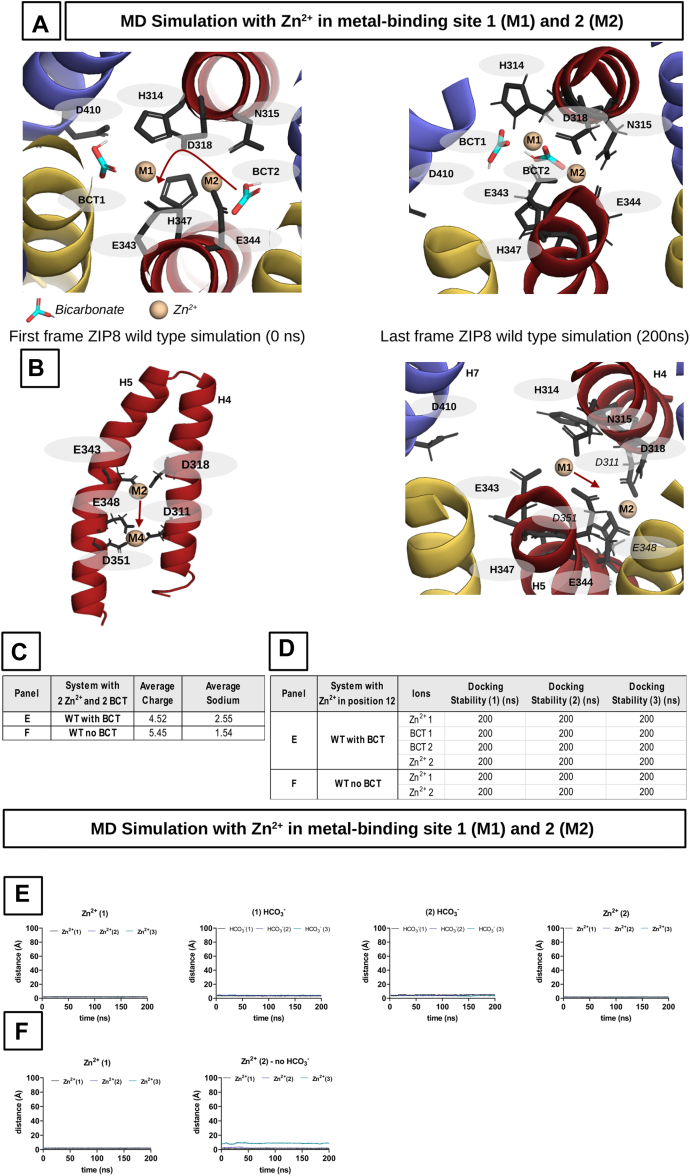
**Molecular dynamics (MD) simulations in a nonelectroneutral system with fully occupied binding sites.** ZIP8-binding site occupied with two Zn^2+^ ions in the presence or absence of two additional HCO_3_^-^ ions. *A*, *left panel*, starting positions for the MD simulations. *Right panel*, status after 200 ns, the end of the simulation. *B*, *left panel*, in the absence of HCO_3_^-^ ions, the Zn^2+^ ion drifts, in one-third of the cases, from position 2 to a new possible binding site (D311, E348, and D351), moving downward. *Right panel*, the Zn^2+^ ion from M1 shifts to a position between the two binding sites M1 and M2. *C*, the table showing the average number of Na^+^ ions in the protein (within a radius of 10 Å around the binding site). *D*, the table complementing the graphs from *C* and *D*, showing the stability of the ions within the protein. *E* and *F*, the trajectories of the individual ions. ZIP8, Zrt/Irt-like protein 8.

To deepen our understanding of how the interactions between HCO_3_^-^ ions and the metal coordinating residues and/or the metal ions themselves can modulate the overall functional activity of ZIP8, we performed a series of molecular dynamics (MD) simulations using our homology-based model of human ZIP8. Based on the identified best molecular docking conformations (Fig. 8*A*), different combinations of HCO_3_^-^ and Zn^2+^ ions were placed in the protein structure in different starting positions (BCT1 and BCT2 for HCO_3_^-^; and M1 and/or M2 for Zn^2+^) and subjected to MD simulations. In an initial simulation, the BCT1, BCT2, M1, and M2 sites were all occupied by the corresponding HCO_3_^-^ and Zn^2+^ ions (Fig. 9). In this configuration, all ions remained within the ZIP8 metal-binding region throughout the entire duration of the simulation. However, the HCO_3_^-^ ion, initially positioned at BCT2, migrated during the equilibration phase to a location between the Zn^2+^ ions placed in M1 and M2 (Fig. 9*A*). Subsequently, to evaluate the influence of HCO_3_^-^ ions in positions BCT1 and BCT2 on the binding stability of the Zn^2+^ in the metal-binding sites M1 and M2, MD simulations were conducted in the absence of HCO_3_^-^. In this scenario, the Zn^2+^ ions showed a higher mobility. It is noteworthy that in one of the three MD simulations, the Zn^2+^ ion from M2 shifts downward and becomes coordinated by a new cluster of charged residues (D311, E348, and D351) (Fig. 9*B*, *left panel*). This observation suggests that these amino acid residues may form an additional metal-binding site, which we termed M4, forming part of a potential metal exit pathway. Meanwhile, the Zn^2+^ ion from M1 shifted to the center between the two binding sites (Fig. 9*B*, *right panel*).

During the course of certain simulations, we have observed cations (Na^+^) from the bulk solvent entering the binding site, indicating a potential charge imbalance. To assess this effect, the average charge and penetration of Na^+^ ions into the metal-binding region were determined throughout the MD simulations (Fig. 9*C*). In the presence of HCO_3_^-^, it was observed that the average charge decreased, whereas the average Na^+^ ion penetration increased. Conversely, in the absence of HCO_3_^-^, the trend was reversed. In our view, these results indicate that the presence of HCO_3_^-^ favors the stability of Zn^2+^ within the metal-binding region, as evidenced by the increased attraction of external positive charges, such as Na^+^ ions. Similarly, in the absence of HCO_3_^-^, the decreased attraction toward external positive charges facilitates the release of Zn^2+^ from the metal-binding region, as evidenced by the transition of Zn^2+^ ions placed in M1 and M2 to new locations within the structure. However, it is remarkable that in none of the experimental configurations, Zn^2+^ and/or HCO_3_^-^ ions move out of the protein structure throughout the MD simulations (Fig. 9, *D*, *E*, and *F* shows the trajectories of the individual ions). Overall, the enhanced stability of Zn^2+^ ions in the presence of HCO_3_^-^ is consistent with the observed increase in affinity in our *in vitro* experiments (Fig. 6).

**Figure 10:**
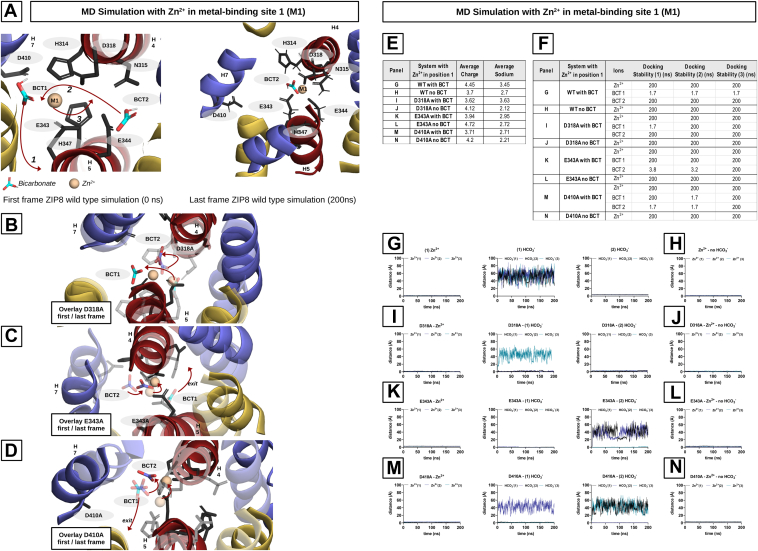
**Molecular dynamics (MD) simulations in an electroneutral system with partially occupied binding sites (M1)**. Binding site 1 of ZIP8 WT and variants D318A, E343A, and D410A occupied by one Zn^2+^ ion in the presence or absence of two additional HCO_3_^-^ ions. *A*, *left panel*, starting positions for the MD simulations. The rearrangement of the ions during the simulation is indicated by *red arrows*. The order of the events is indicated by numbers 1 to 3. *Right panel*, state after 200 ns, the end of the simulation. *B*, overlay of the D318A variant with the first (helices and ions faded out) and last frame. Both HCO_3_^-^ ions stay stable within the binding site. *C* and *D*, overlay of the E343A and D410A variants with first (helices and ions faded out) and last frames. Instability in the BCT2 area is visible. HCO_3_^-^ placed in position BCT2 leaves. *E*, the table showing the average number of Na^+^ ions in the protein (within a radius of 10 Å around the binding site). *F*, the table complementing the graphs from *G* to *N* and showing the stability of the ions in the protein. None of the ions left ZIP8 during the simulations. *G*, *I*, *K*, and *M* , trajectories of individual ions from the simulations with one Zn^2+^ ion and two HCO_3_^-^ ions (WT and variants). *H*, *J*, *L*, and *N*, trajectories of the Zn^2+^ ions from the simulations in the absence of HCO_3_^-^ ions (WT and variants). ZIP8, Zrt/Irt-like protein 8.

It has been proposed that ZIP8-mediated transport is electroneutral (41, 44, 45, 61, 62). Therefore, we decided to perform MD simulations on a configuration comprising a single Zn^2+^ ion in position M1 and two HCO_3_^-^ ions in positions BCT1 and BCT2. In this scenario, the HCO_3_^-^ ion in position BCT1 exits the protein within the first few nanoseconds, whereas the Zn^2+^ ion moves to a central position between M1 and M2, and the HCO_3_^-^ ion in position BCT2 moves to a position nearby BCT1 (Fig. 10*A*). In this new configuration, the ions remained stable throughout the remainder of the simulation. Consistent with the preceding simulations (Fig. 9), these results indicate that the most stable configurations within binding sites M1 and M2 are achieved when the ratio of HCO_3_^-^ to Zn^2+^ is 1:1.

Our *in vitro* experiments showed that replacing the charged residues D410, E343, and D318 with alanine resulted in a loss of function (Fig. 3). Interestingly, in the case of the D410A and E343A variants, the function could be rescued by HCO_3_^-^ (Fig. 6). To validate the hypothesis that HCO_3_^-^ could rescue the function of these variants by providing the missing negative charge, we performed MD simulations using the same electroneutral system configuration (*i*.*e*., Zn^2+^ ion in position M1 and two HCO_3_^-^ ions in positions BCT1 and BCT2) with the loss-of-function ZIP8 variants D410A, E343A, and the functionally inactive variant D318, as a negative control. In contrast to WT ZIP8, for the D410A and E343A variants, the HCO_3_^-^ ion in position BCT2 exits the protein within the first few nanoseconds, whereas the Zn^2+^ ion moves to a central position between M1 and M2, and the HCO_3_^-^ ion in position BCT1 remains nearby the initial position (Fig. 10*B*).

It is noteworthy that the HCO_3_^-^ ion in position BCT2 exits in the M4 direction, indicating that HCO_3_^-^ can exit the protein in two ways: straight out of M1 (Fig. 10*A*) or through the M2–M4 pathway, the potential exit route that we already observed for the Zn^2+^ ions in the previous simulations (Fig. 9*B*, *left panel*). Furthermore, given that residues D410 and E343 are part of M1, the stability of the HCO_3_^-^ ion in BCT1 indicates that, in this specific context, it compensates for the loss of negative charge because of the mutations. This is consistent with our interpretation of the functional rescue by HCO_3_^-^ observed in the *in vitro* experiments (Fig. 6).

Surprisingly, for the D318A variant, none of the HCO_3_^-^ ions leave the metal-binding region during the simulations. It is noteworthy that, instead of exiting the protein, the HCO_3_^-^ ion in position BCT1 shifts toward the region previously occupied by the D318 side chain in the WT ZIP8. The Zn^2+^ ion also relocates to a central position between M1 and M2 but in a closer proximity to residues E343 and E344. In turn, the HCO_3_^-^ ion in position BCT2 still moves nearby the BCT1 initial position. In our view, this simulation provides further evidence that HCO_3_^-^ ions can compensate for the loss of charge because of the mutations. It also suggests that the closer proximity of the Zn^2+^ ion to residues E343 and E344 may result in a stronger interaction with this region of the metal-binding site. This could potentially prevent the release of the Zn^2+^ ion and lead to the complete impairment of the metal transport observed in our *in vitro* experiments with this variant (Fig. 3).

In addition, simulations were performed for WT ZIP8 and the E343A, D410A, and D318A variants, in the absence of HCO_3_^-^ and the Zn^2+^ ion in position M1. Interestingly, the Zn^2+^ ions behaved exactly as observed in the simulations in the presence of HCO_3_^-^, relocating to a central position between M1 and M2, where they remained throughout the simulation period (Fig. 10*F*). The average charge and Na^+^ ion penetration into the metal-binding region were determined for all these simulations (Fig. 10*E*). As observed previously, Na^+^ ion penetration was higher in the configurations containing HCO_3_^-^ ions, except for the functionally inactive D318A variant (Fig. 10, *G* to *N* shows the trajectories of the individual ions).

We believe that this reflects the ability of HCO_3_^-^ ions to promote the stability of Zn^2+^ within the metal-binding region observed as enhanced affinity for Cd^2+^ ions in the presence of HCO_3_^-^ in our *in vitro* experiments (Fig. 6). Average charge determinations show that, in contrast to WT ZIP8, variants E343A, D410A, and D318A exhibit a higher average charge in the absence of HCO_3_^-^ ions (Fig. 10*E*). Visual inspection of the simulations with these variants reveals that they have a wider metal-binding region, which allows greater penetration of charged ions into the region. Interestingly, the permeability of the binding site to charged ions is restored in the presence of HCO_3_^-^ ions, as evidenced by the reduced average charges calculated for all the mutant variants.

**Figure 11:**
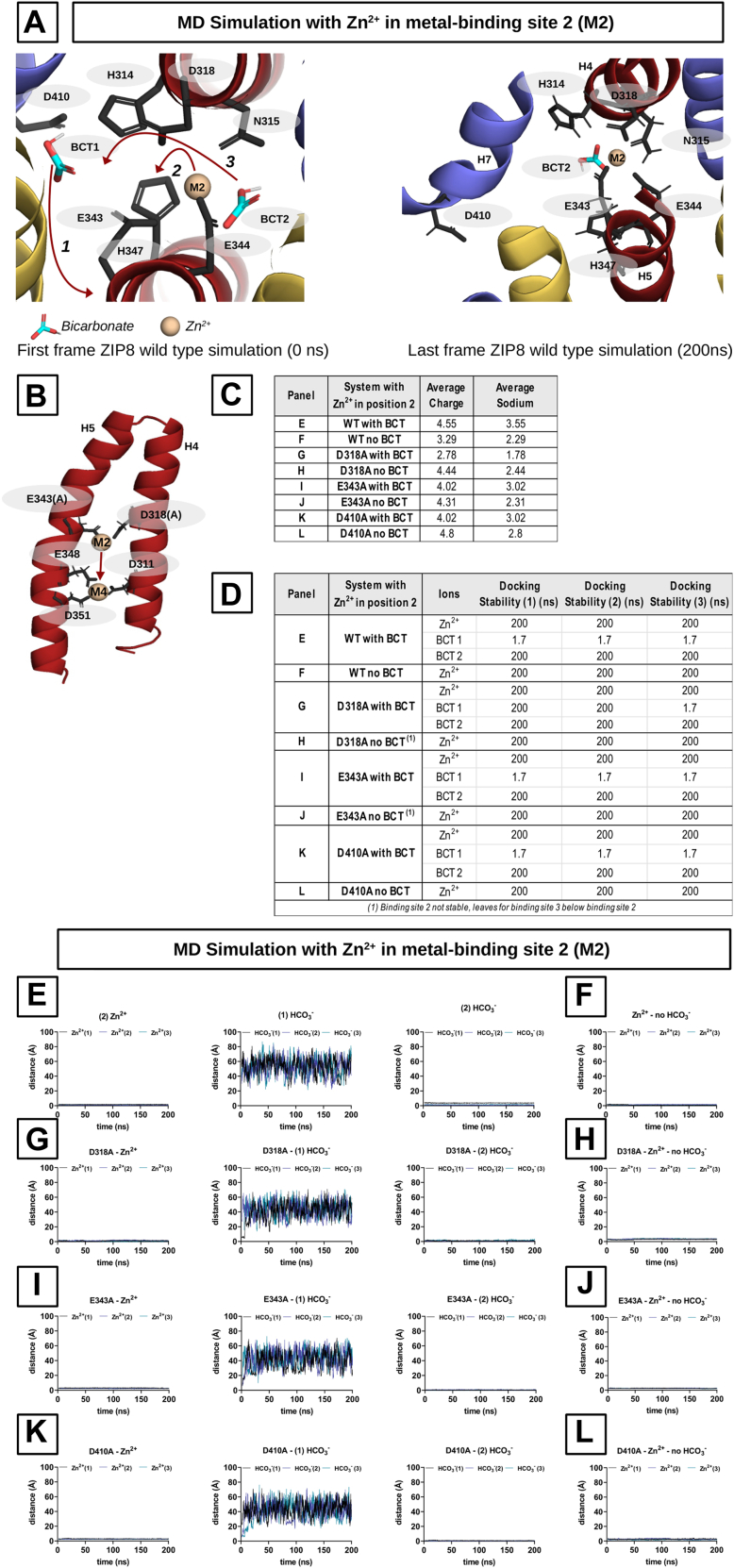
**Molecular dynamics (MD) simulations in an electroneutral system with partially occupied binding sites (M2)**. Binding site 2 of ZIP8 WT and variants D318A, E343A, and D410A occupied by one Zn^2+^ ion in the presence or absence of two additional HCO_3_^-^ ions. *A*, *left panel*, starting positions for the MD simulations. The rearrangement of the ions during the simulation is indicated by *red arrows*. The order of the events is indicated by numbers 1 to 3. *Right panel*, state after 200 ns, the end of the simulation. *B*, Zn^2+^ ion drifts into the newly identified binding site (D311, E348, and D351), as observed in Figure 9*C*. The table showing the average number of Na^+^ ions in the protein (within a radius of 10 Å around the binding site). *D*, the table complementing the graphs from *E* to *L* and showing the stability of the ions in the protein. None of the ions left ZIP8 during the simulations. *E*, *G*, *I*, and *K*, trajectories of individual ions from the simulations with one Zn^2+^ ion and two HCO_3_^-^ ions (WT and variants). *F*, *H*, *J*, and *L*, trajectories of the Zn^2+^ ions from the simulations in the absence of HCO_3_^-^ ions (WT and variants). ZIP8, Zrt/Irt-like protein 8.

Finally, we assessed whether placing the Zn^2+^ in position M2 instead of M1 would affect the outcome of the simulations. To do this, all the above simulations were repeated with the initial configuration consisting of a single Zn^2+^ ion in position M2 and two HCO_3_^-^ ions in positions BCT1 and BCT2. The ion transition patterns (Fig. 11*A*), the average charge and Na^+^ ion penetration (Fig. 11*C*), and the docking stability of the different ions (Fig. 11*D*) were in agreement with the results of the previous simulations (Fig. 10). However, when the simulations were performed in the absence of HCO_3_^-^ ions, significant differences were observed in the D343A and D318A variants. The Zn^2+^ ion, instead of moving to a central position between M1 and M2, shifted to the previously described M4 (Fig. 9), consisting of residues D311, E348, and D351, where it remained stably bound throughout the remaining simulation time (Fig. 11*B*). WT ZIP8, D343A, and D318A exhibited identical behavior in the presence of HCO_3_^-^ ions, whereas in the absence of HCO_3_^-^ ions, the stability of the Zn^2+^ ion in the metal-binding region (*i*.*e*., M1 and M2) was compromised for the mutant variants.

The conducted MD simulations overall support the interpretation of the functional rescue by HCO_3_^-^ observed in the *in vitro* experiments (Fig. 6). Furthermore, the transition of Zn^2+^ ions from M2 to M4, which could potentially constitute part of the exit pathway, as a consequence of a local alteration of the electrostatic equilibrium (*i*.*e*., in this case induced by the introduced mutations), illustrates a potential mechanism by which metal ions could be released from the metal-binding region. In this regard, the effect of HCO_3_^-^ ions in preventing the M2–M4 transition provides further evidence of their role as a modulator of the ZIP8-mediated metal transport process.

### Zn^2+^, HCO_3_^-^, and HSeO_3_^-^ cotransport by ZIP8

Metal ion transport by ZIP8 and its closely related paralog ZIP14 has been found to be electroneutral, and it has been proposed that this is due to cotransport of HCO_3_^-^ or other ionic species (41, 44, 45, 59, 61, 62, 69). One proposal was that ZIP8 mediates the transport of an electroneutral complex containing three ions: Zn^2+^, HCO_3_^-^, and HSeO_3_^-^ (63). However, to date, there is no experimental evidence for the cotransport of HCO_3_^-^ and/or HSeO_3_^-^.

**Figure 12:**
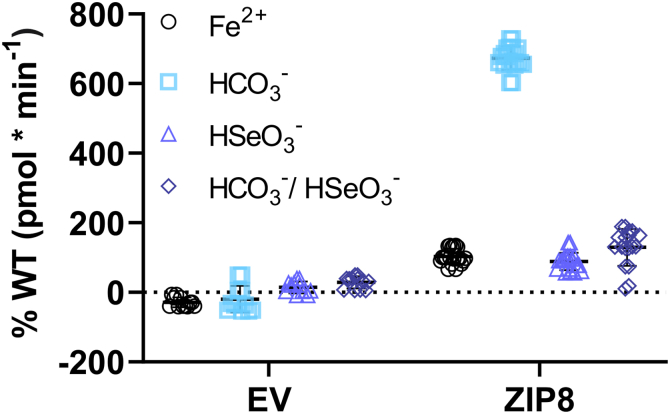
**Effect of extracellular selenite (HSeO_3_^-^) on iron transport by human ZIP8**. Radiolabeled ^55^Fe^2+^ uptake in the absence and presence of extracellular 25 mM NaHSeO_3_, 25 mM NaHCO_3_, or an equimolar combination of both (*i*.*e*., 12.5 mM). Compared with the control condition, extracellular NaHSeO_3_ alone does not stimulate the ^55^Fe^2+^ uptake, whereas extracellular HCO_3_^-^ increases ^55^Fe^2+^ uptake about sixfold. Conversely, in the presence of both HCO_3_^-^ and NaHSeO_3_, ^55^Fe^2+^ uptake is stimulated only 1.5-fold. N = 12 to 24. ZIP8, Zrt/Irt-like protein 8.

In order to assess whether the HCO_3_^-^–HSeO_3_^-^ complex can be cotransported together with the metal ion by ZIP8, radiolabeled ^55^Fe^2+^ transport was measured in the presence of extracellular HCO_3_^-^ and HSeO_3_^-^ alone or in combination (Fig. 12). Interestingly, the presence of HSeO_3_^-^ alone had no stimulatory effect on Fe^2+^ transport in cells overexpressing ZIP8, whereas HSeO_3_^-^ abolished the stimulation induced by HCO_3_^-^. No significant differences were observed for the different experimental conditions with the EV-transfected cells. These results suggest that HSeO_3_^-^ is not transported by ZIP8 but that it competes with HCO_3_^-^, thereby preventing its stimulatory effect on ZIP8-mediated Zn^2+^ transport.

## Discussion

In the present study, we have generated a homology-based structural model for human ZIP8 and identified key residues for divalent metal ion binding, substrate selectivity, and transport turnover rate using a variety of functional assays. Furthermore, we have investigated the potential influence of HCO_3_^-^ on the functional activity of ZIP8 using a combination of functional experiments and molecular docking. Finally, a series of MD simulations were performed to gain insight into the molecular processes occurring during ZIP8-mediated metal ion transport in the presence of HCO_3_^-^.

### Binuclear metal center

Our structural model of human ZIP8 was based on the crystal structure of the homologous prokaryotic BbZIP, which contains a binuclear metal-binding center comprising M1 and M2 (52). Metal ion soaking and mutagenesis in BbZIP, including a triple M2 mutant (N178A/D208A/E240A), showed that disrupting M2 does not alter the overall structure or M1 metal coordination, suggesting M2 is dispensable in BbZIP (53). Similarly, in human ZIP4, mutations targeting M1 (H507A/H540A/H536) and M2 (N508A/E537A) supported the idea that M2 is not essential for transport but contributes to maximal activity (52, 53). This is further supported by findings in ZIP1, ZIP2, and ZIP9, where a lysine in the M2 center likely abolishes M2 function (49).

In contrast, our mutagenesis studies with human ZIP8 suggest a different role for M2. Point mutations in M2 residues N315 and E344, as well as in bridging residue D318, led to complete loss of Fe^2+^ and Cd^2+^ transport, indicating these residues are critical for metal coordination and essential for function. This implies that, unlike in other ZIPs, M2 integrity is necessary for ZIP8 transport function or that the structure of this binding site is less tolerant to local electrostatic disruptions.

Conversely, mutations in M1 residues (H314, E343, and H347) retained Cd^2+^ and Fe^2+^ transport capacity, with the exception of E343, whose variants could only transport Cd^2+^ (Fig. 3). We also examined D410 as a potential M1 metal coordination site. Interestingly, the equivalent residues in BbZIP and ZIP4 (E276 and D604) were recently implicated in an additional M3-binding site located near the transport exit (70). Mutations at position D410 abolished Fe^2+^ transport and markedly reduced Cd^2+^ transport. These findings support the proposed involvement of D410 as a coordinating residue in M1 or M3, or at least, highlight its importance in the expanded metal selectivity of ZIP8 (70).

### Regulation by pH and metal transport pathway

One potential factor that may contribute to the observed differences in M1–M2 function among other ZIP members and ZIP8 is pH regulation. While BbZIP (47), ZIP2 (48, 49) and ZIP4 (46) have been shown to be positively modulated by extracellular favorable H^+^ gradients or even to directly couple substrate transport to H^+^, ZIP8 has been shown to function optimally at neutral pH (71) and to be stimulated by extracellular favorable HCO_3_^-^ gradient (44, 45), as evidenced by the results presented herein (Figs. 6 and 7). The opposite pH regulation suggests that the ZIP8 metal transport pathway, the role of the M1- and M2-binding sites, and/or the overall architecture of the binuclear center must present meaningful differences compared with BbZIP, ZIP2, or ZIP4. Related to this, two distinct metal transport pathways have been proposed for BbZIP. The first postulates that M2 functions as a regulatory site capable of modulating the properties of M1 by affecting the geometry and charge of metal-binding residues on M1, which is considered the primary metal transport site. In this scenario, the transported metal ion enters and exits the binuclear center *via* M1. In the second scenario, both M1 and M2 are part of the metal transport pathway. The metal first binds to the M2 site and then transfers to the M1 site before being released into the cytoplasm (72). Since M2 was found not to be essential for the transport function of BbZIP, this suggests that the first transport pathway is the more likely one, in which M1 is the primary site and M2 is complementary. But for ZIP8, M2 is essential for the transport function (Fig. 3). Therefore, it can be proposed that the second transport pathway is a better fit for ZIP8, since both M1 and M2 are necessary for functional activity, which requires metal transport through M2. It remains to be validated whether a difference in the metal transport pathway is responsible for the observed difference in pH modulation between BbZIP and ZIP8, or whether it is a consequence of differences in the binuclear metal center architecture, particularly in terms of amino acid residue composition. Furthermore, it is conceivable that in the case of ZIP8, M2 represents the primary transport site, whereas M1 serves as an auxiliary function. Unfortunately, the homology-based 3D structure presented here for human ZIP8 (Fig. 1*A*) shows an inward–open conformation, which precludes the possibility of addressing the aforementioned questions based on our model.

### Amino acid residues contributing to metal transport kinetics

Our transport kinetic studies with human ZIP8 and the functionally active variants (*i*.*e*., H314A, H347A, H347N, E343A, E343Q, D410A, and D410N) allowed us to gain a deeper understanding of the function of each of these residues in M1. Our findings indicate that H347 and H314 are not necessary for Fe^2+^ or Cd^2+^ coordination, as the variants H314A and H347N showed no significant loss of transport function (Fig. 3). Interestingly, for the H347A variant, the affinity for Fe^2+^ decreased fourfold, whereas the *V*_max_ increased threefold (Fig. 4). The location of the H347 residue on the exit pathway from M1 to the intracellular milieu suggests that it may play a role in certain structural rearrangements necessary for metal release from the binuclear metal center, thus affecting the turnover rate of metal transport. Consistent with this, the H540 residue in human ZIP4 (equivalent to H347 in human ZIP8) was found to exhibit more than a twofold increase in transport activity when mutated to alanine, and it was proposed that this residue plays a role in controlling the rate of metal release and/or conformational switching of the transporter during the transport cycle (50).

Interestingly, in our experiments with human ZIP8, the E343A, E343Q, D410A, and D410N variants were not able to transport Fe^2+^. Conversely, the *K*_*m*_ values of these variants for Cd^2+^ transport were in a similar range to those of WT ZIP8 (Fig. 4). However, the *V*_max_ values for Cd^2+^ transport by these variants were approximately half of that observed in WT ZIP8. Therefore, we interpret that the impaired Fe^2+^ transport and reduction of the *V*_max_ to half for Cd^2+^ transport by the E343 and D410 variants indicates that these residues are key for M1 functional activity. Moreover, from these results, it can be concluded that M1 is only necessary for maximal transport capacity of Cd^2+^, whereas it is required for Fe^2+^ transport, since M2 is not able to support Fe^2+^ transport if the integrity of M1 is compromised.

As for residue E343, in the corresponding position, there is a highly conserved histidine residue for almost all ZIP family members, whereas both ZIP8 and ZIP14 have a glutamic acid residue at this position. This led to the hypothesis that E343 is responsible for the extended substrate selectivity that is characteristic of both human ZIP8 and ZIP14 (65, 66). Our results provide direct experimental evidence to support this hypothesis. Moreover, the effect of E343 variants on Cd^2+^ transport kinetics, with a substrate affinity two to three times lower than that of WT ZIP8, further supports the role of this residue in metal coordination in the binuclear metal center. Interestingly, in a recent report, functional evaluation of the E343A and E343H human ZIP8 variants showed that Mn^2+^ and Cd^2+^ transport were completely abolished compared with WT (24). These results further support the hypothesis that E343 is a key residue for the expanded metal selectivity of ZIP8 and ZIP14, which includes Mn^2+^ as a specific substrate (Fig. 4) (18).

Regarding the D410 variants, they appear to abolish Fe^2+^ transport and significantly decrease the *V*_max_ of Cd^2+^ transport without altering the *K*_*M*_ (Fig. 4). The corresponding residues in BbZIP and human ZIP4 are E276 and D604, respectively. Both residues have been proposed to be part of the metal coordination site M3, which is defined as an additional metal-binding site located at the exit of the metal transport pathway (55, 70). Interestingly, the E276A mutant in BbZIP did not affect the functional activity, suggesting that the M3 site is functionally dispensable. In contrast, the D604A mutant in human ZIP4 completely abolished functional activity, suggesting additional mechanistic roles (70). Consequently, the authors proposed that metal release from the binuclear metal center of BbZIP into the cytoplasm can occur through alternative exit pathways and thus is not completely dependent on E276, whereas for ZIP4, the equivalent D604 residue is critical to prevent transported metal ions from being pulled back to the high-affinity binuclear metal center (70). In human ZIP8, D410 variants lose the ability to transport Fe^2+^ and probably also the transport of Cd^2+^ bound to M1, since our results suggest that M1 is inactive upon mutation of this residue. Therefore, it appears to be either part of the M1 site, although D410 variants did not alter the apparent *K*_*M*_ for Cd^2+^, or at least responsible for mediating the release of the metal ion from M1. Since there is a reduced but still significant transport activity for the D410 variant, in contrast to the observations made with the corresponding BbZIP and ZIP4 variants, it is possible that D410 plays a different role in ZIP8, or alternatively, that there are different intracellular exit pathways in human ZIP8. Multiple alignments between ZIP family members show that negatively charged amino acids, such as glutamic acid or aspartic acid (Fig. 1*C*), are frequently found at this position located in TMH7, suggesting a conserved transport function for these residues. Consistent with this concept, in a previous work from our group, the human ZIP2 E276A variant, which carries a substitution of this residue at the corresponding position, showed an almost completely abolished activity, which was restored to almost WT levels in the E276Q mutant. Therefore, we proposed that, in addition to coordinating metal ions, E276 in ZIP2 could act as an intracellular gate (49). Taken together, the observations made with BbZIP, ZIP2, and ZIP4 indicate that the presence of a negatively charged amino acid residue at this site within TMH7 affects the transport function and metal release from M1. However, the distinct observations for the ZIP8 D410 variants suggest a different metal transport pathway for human ZIP8.

### Amino acid residues contributing to substrate selectivity

Our metal competition experiments showed that residues H314, E343, and D410 are crucial for the substrate selectivity of human ZIP8. While H314 likely does not directly coordinate metals in M1, its position along the entry pathway suggests a role as a selectivity filter. Supporting this, the H314A mutant showed altered substrate preference, including Ba^2+^ binding (Fig. 5, *A* and *E*). This histidine residue is conserved across most ZIPs (Fig. 1*C*). In ZIP4, the equivalent H507A mutation reduces activity by almost half (50), consistent with ZIP8 H314A behavior (Fig. 3). In ZIP2, mutating the corresponding H175 to alanine abolishes activity, suggesting a direct role in metal coordination there (49). Similarly, ZIP2 mutants of F269—another residue on the access path—like F296A, showed altered substrate selectivity (*e*.*g*., Mn^2+^ binding) with unchanged *K*_*M*_ values. This supports the concept that aromatic residues like histidine or phenylalanine help define substrate size selectivity by limiting access to the binuclear center.

E343 has been proposed to explain the extended metal ion range of ZIP8 (65, 66). Though E343 mutants cannot transport Fe^2+^ (Fig. 3), they still bind it like WT (Fig. 5*L*). By contrast, D410 mutants neither transport nor bind Fe^2+^ or Mn^2+^ (Fig. 5, *I* and *J*), indicating D410 is essential for extended selectivity. However, since other ZIPs with a conserved aspartate at this position do not transport Fe^2+^ or Mn^2+^ (Fig. 1*C*), additional factors must contribute. Both E343 and D410 are needed for M1 site integrity, critical for transporting larger metals like Fe^2+^. While classical ZIP substrates (Zn^2+^, Cd^2+^) can use either M1 or M2, Fe^2+^ and Mn^2+^ transport depends on an intact M1, with E343 and D410 as key determinants in ZIP8.

### Me^2+^–HCO_3_^-^ cotransport mechanism

In the present work, it was observed that HCO_3_^-^ increases the affinity of ZIP8 for its substrates Cd^2+^ and Fe^2+^ (Fig. 6). The calculated *K*_*M*_ value for Cd^2+^ in the presence of 25 mM HCO_3_^-^ was 0.36 μM, fourfold lower than in the absence (*i*.*e*., *K*_*M*_ = 1.6 μM). Similarly, the calculated *K*_*M*_ value for Fe^2+^ in the presence of 25 mM HCO_3_^-^ was 0.84 μM, sixfold lower than in the absence (*i*.*e*., *K*_*M*_ = 5.18 μM). These results are in good agreement with a previous study using microinjected *Xenopus laevis* oocytes as expression system, in which under similar conditions (*i*.*e*., 25 mM HCO_3_^-^), the *K*_*M*_ values calculated for Cd^2+^ and Fe^2+^ were 0.48 and 0.16 μM, respectively (59). Consistent with these findings, in another study, Cd^2+^ and Mn^2+^ transport by Madin–Darby canine kidney cells transiently transfected with ZIP8 was severalfold higher in the presence of HCO_3_^-^ (44). Collectively, these findings suggest a role for HCO_3_^-^ in the transport mechanism driving the metal transport function of ZIP8.

The present study provides the first direct evidence of Zn^2+^–HCO_3_^-^ cotransport (Fig. 7). Intracellular pH measurements revealed that WT ZIP8 overexpression results in a significant increase in intracellular pH because of Zn^2+^–HCO_3_^-^ cotransport. Moreover, molecular docking studies have identified two potential HCO_3_^-^-binding sites near the binuclear metal center, designated as BCT1 and BCT2 (Fig. 8). Residues N315 and E344 (M2) appear to be involved in HCO_3_^-^ binding to BCT2, forming H-bonds with the HCO_3_^-^ ion. However, since mutation of these residues is not compatible with functional activity (Fig. 3), whether these residues affect the Zn^2+^–HCO_3_^-^ cotransport could not be assessed. In contrast, residue D410, which appears to be involved in HCO_3_^-^ binding in BCT1 (*i*.*e*., forming H-bonds with the HCO_3_^-^ ion), retained its functional activity upon mutation (Fig. 3), despite its substantial impact on M1 function. The introduction of mutations at position D410 resulted in the complete loss of the ability to transport Fe^2+^, whereas the *V*_max_ of Cd^2+^ transport was significantly reduced without altering the *K*_*M*_ (Fig. 4). It is unclear whether this effect on the ZIP8 transport function is a consequence of the loss of the negative charge provided by D410 side chain because of the mutation to A or N, or of the impairment of BCT1 function. It is noteworthy that in the presence of HCO_3_^-^, the D410 variants demonstrate a recovery of their functional activity (Fig. 6), suggesting that HCO_3_^-^ may provide the missing metal coordination site that has been lost because of the mutation. Interestingly, when measuring the effect of D410 mutations on intracellular pH changes induced by Zn^2+^–HCO_3_^-^ cotransport, no changes in the intracellular pH were observed in contrast to WT ZIP8 (Fig. 7). We proposed that in this scenario, a Zn^2+^–HCO_3_^-^ complex could be transported, resulting in a reduced availability of free HCO_3_^-^ to modulate intracellular pH levels. Nevertheless, our MD simulations (Figs. 10 and 11) show that the D410A variant has a distinct ion transition pattern compared with WT ZIP8. In this variant, the HCO_3_^-^ ion in BCT1 does not leave the protein structure but remains near the Zn^2+^ ion located between M1 and M2, forming a stable complex throughout the entire simulation. It can therefore be proposed that this evidence supports the idea of a more stable Zn^2+^–HCO_3_^-^ complex, which may be the consequence of the local electrostatic imbalance resulting from the loss of negative charge because of the D410A mutation. However, the question of whether the Zn^2+^–HCO_3_^-^ complex is released into the intracellular milieu remains unanswered, as this was not resolved during the simulation period. In support of this hypothesis, similar observations were also made with the E343A variant in both *in vitro* experiments (Figs. 6 and 7) and MD simulations (Fig. 9).

As previously stated, BCT1 interacts with residues situated in the M1 metal coordination site. Particularly, HCO_3_^-^ is located between H347, the negatively charged D410, and the metal ion Zn^2+^. As shown in our *in vitro* experiments (Fig. 4, *B* and *D*), H347 appears to have a major effect on the turnover rate of ZIP8 transport. Consequently, electrostatic interactions with BCT1 could potentially modulate the transport activity of ZIP8. In agreement with this, in the experiments performed with the H347A variant in the presence of HCO_3_^-^ resulted in a marked reduction of the transport turnover rate, which was several times higher than that of WT in the absence of HCO_3_^-^ (Fig. 6*C*). It is also noteworthy that the repulsion between the negative charges of BCT1 and the neighboring side chains of the metal coordinating residues D410 and E343 could potentially trigger structural changes with an impact on the transport turnover rate. In support of this hypothesis, the variants of these residues D410A, D410N, E343A, and E343Q showed a recovery of their decreased transport turnover rates in the presence of HCO_3_^-^ (Fig. 6*C*). In our view, this is consistent with the potential interplay between BCT1 and those residues in the context of WT ZIP8 turnover rate modulation.

### Me^2+^–HCO_3_^-^ stoichiometry

An interesting finding of our MD simulations is that the most stable configurations are achieved when the Zn^2+^ and HCO_3_^-^ ions are present in a 1:1 ratio (Fig. 9), which may indicate an electrogenic transport process. This would contrast with previous studies, which have proposed that ZIP8-mediated transport is electroneutral, postulating that transport occurs as a complex of Zn^2+^–(HCO_3_^−^)_2_ (61). In line with this hypothesis, in another study, it has been proposed that the electroneutral complex transported by ZIP8 could also be constituted by three distinct ions, namely Zn^2+^–(HCO_3_^−^)–(HSeO_3_^−^) (63). However, our results on the transport of selenite (HSeO_3_^−^) across ZIP8 did not support the proposal that it can be cotransported (Fig. 12).

Consistent with the electrogenic transport mechanism, the initial configuration consisting of Zn^2+^ ions in both M1 and M2 and HCO_3_^−^ ions in both BCT1 and BCT2 was observed to remain stable throughout the entire MD simulation period (Fig. 9). In contrast, the initial configuration constituted by a single Zn^2+^ ion in either M1 or M2 and HCO_3_^−^ ions in both BCT1 and BCT2 (*i*.*e*., electroneutral) resulted in the HCO_3_^−^ ion in position BCT1 leaving the protein. Moreover, the HCO_3_^−^ ion in position BCT1 was observed to relocate near the Zn^2+^ ion located between M1 and M2 (Figs. 10 and 11). These results indicate that an electroneutral complex would not be stably bound within the binuclear binding center in the inward-facing conformation, thus favoring substrate release into the intracellular milieu. However, the binding to the outward–open conformation must also be evaluated to determine whether electroneutral complex binding would be possible.

It is also noteworthy that, in the absence of HCO_3_^−^ ions, transitions of one of the two Zn^2+^ ions out of the binuclear metal center were observed (Fig. 9*B*). In this context, both Cd^2+^ and Fe^2+^ transport was observed in the zero HCO_3_^−^ condition (Fig. 7), suggesting that electrogenic transport through ZIP8 could be possible. However, the presence of nominal HCO_3_^-^ because of the exogenous HCO_3_^-^ generated from dissolved CO_2_ present in the air, as well as the CO_2_–HCO_3_^-^ generated by cellular metabolism, does not allow a proper assessment of this condition. Therefore, further studies will be necessary to determine whether HCO_3_^-^-independent metal ion transport through ZIP8 is possible.

Finally, the possibility that the M4 site, identified in our current work, may act as a cation-bound regulatory site should be considered. In our MD simulations, the acidic amino acid side chains of the M4 site were assigned a deprotonated state, reducing the overall charge of the neighborhood of the substrate-binding site and potentially repelling or destabilizing negatively charged HCO_3_^-^ molecules. However, it is possible that the M4 site is permanently occupied by a divalent metal ion or protons, thereby stabilizing an overall neutral Zn^2+^–(HCO_3_^-^)_2_ complex in the M1–M2 and BCT1–BCT2 sites. Electroneutral transport could then be realized by a mobile neutral Zn^2+^–(HCO_3_^-^)_2_ complex translocated across the membrane, whereas the nontransported divalent metal in the M4 site could play a regulatory role. This arrangement is partially supported by our MD simulations, which show that two Zn^2+^ and two HCO_3_^-^ ions can be stably supported by the binding sites.

### New metal-binding site—M4

As alluded to above, our MD simulations have revealed the existence of an additional putative substrate-binding site, which we have named M4. This site is formed in human ZIP8 by D311 (TMH4), E348 and D351 (TMH5), which are conserved in most human ZIPs (Fig. 1C), except for ZIP1-3 (*SLC39A1-3*) and ZIP9 (*SLC39A9*). Similarly, for ZIP11 (*SLC39A11*), only the D311 residue is not conserved. Furthermore, this additional metal-binding site has not been identified in BbZIP structures, as the corresponding residues in BbZIP typically possess hydrophobic characteristics and are therefore unlikely to interact with metal ions. The presence of three acidic residues suggests that the M4 site has a high affinity for cations. As shown by the results of our MD simulations, M4 can form a stable bond with a Zn^2^. However, the precise role of M4 consisting of residues in TMHs 4 and 5 in ZIP8 metal transport activity remains unclear. Given that they are located underneath the well-described binuclear metal center, it is unexpected that they serve as an additional metal-binding site in the outward-facing conformation. Therefore, based on the results of our MD simulations, which reveal transition of the Zn^2+^ ion from M2 to M4 (Figs. 9*B* and 11*B*), it can be postulated that M4 may play a role in the transfer of the metal to the intracellular milieu. An alternative hypothesis just mentioned above is that M4 may function as an additional regulatory site. These open questions warrant further investigation to determine the validity of M4 and its precise role in the ZIP8 transport function.

## Conclusion

In the present study, we generated the first 3D homology model of human ZIP8, allowing us to identify key residues forming the binuclear metal center: H314 (TMH4), E343 (TMH5), H347 (TMH5) in M1; N315 (TMH4), E344 (TMH5) in M2; D318 (TMH4) bridging M1 and M2; and D410 (TMH7) interacting with M1. Site-directed mutagenesis and functional studies revealed their roles in metal transport kinetics and substrate selectivity. Mutating N315, E344, or D318 completely abolished the function, highlighting the central role of M2, unlike in other ZIP family members, which rely on M1 for this task. In contrast, mutations in H314, E343, or D410 preserved activity but often altered substrate specificity or kinetics. Specifically, H314 influenced substrate selectivity; E344 and D410 were essential for Fe^2+^ and Mn^2+^ transport, a distinctive feature for ZIP8 and ZIP14; and H347 modulated transport turnover rates. Overall, M1 residues support accessory functions, such as maximizing transport rates and refining substrate selectivity, while being less critical for Zn^2+^ and Cd^2+^ transport.

We also present the first direct evidence for Zn^2+^–HCO_3_^-^ cotransport by human ZIP8. Functional assays, molecular docking, and MD simulations identified two potential HCO_3_^-^-binding sites within the binuclear metal center, BCT1 and BCT2, that affect metal transport. HCO_3_^-^ enhanced metal ion affinity, likely through additional metal coordination at these sites, with the most stable configuration observed at a 1:1 Zn^2+^–HCO_3_^-^ ratio. Deviations from this ratio triggered ion shifts within ZIP8, suggesting that electrostatic perturbations influence key transport steps. MD simulations also revealed a novel metal site, M4 (D311, E348, and D351), associated with these transitions induced by unbalanced Zn^2+^–HCO_3_^-^ ratios or mutagenesis.

Given the observed M2 to M4 metal transitions and the proximity of M4 to the intracellular milieu, M4 may participate in the divalent metal ion exit pathway or act as a regulatory site permanently occupied by a divalent metal ion (*e*.*g*., Zn^2+^). In the latter case, M4 occupancy could provide complementary electrostatic charge to facilitate transport of an electroneutral complex consisting of one Zn^2+^ ion and two HCO_3_^-^ ions.

This work advances understanding of the 3D structure of human ZIP8, identifies residues essential for transport, and elucidates elements of the Zn^2+^–HCO_3_^-^ cotransport mechanism. Outstanding questions include defining the exact function of M4, validating the proposed HCO_3_^-^-binding sites, clarifying differences between ZIP8 and other ZIP family members (*e*.*g*., reverse pH modulation), and delineating the precise roles of M1 and M2 in the transport cycle. Resolving these issues will require experimentally determined ZIP8 structures in multiple configurations to map the complete transport cycle.

## Experimental procedures

### Human ZIP8 (*SLC39A8*) single-point mutation generation

The following amino acid residues in the human ZIP8 protein-coding sequence were mutated by PCR amplification: Ser-145, His-314, His-347, Asn-315, Asp-318, Asp-410, Glu-343, and Glu-344. The coding sequence of human ZIP8 inserted into the pIRES2-DsRED-Express vector was used as a template for the site-directed mutagenesis. Primers were designed to introduce the following mutations: Ser to Ala; His to Ala, Asp, or Arg; Asn to Ala; Asp to Asn, Ala, or Glu; Glu to Gln, Ala. The resulting PCR products were used to transform DH5-alpha–competent cells (Thermo Fisher) by the heat-shock method. Transformed cells were selected on LB agar plates supplemented with kanamycin (30 μg/ml). DNA was isolated from single clone colonies and sequenced (Microsynth AG) to validate the correct introduction of the designed mutations. Positive clones were expanded and used in subsequent functional studies.

### Cell culture

Human embryonic kidney 293T (HEK293T) cells were obtained from the American Type Culture Collection. HEK293T cells were cultured in a cell culture incubator providing constant environmental conditions of 5% CO_2_, 95% humidity, and 37°C. The medium used to culture the cells was Dulbecco’s modified Eagle's medium (Sigma–Aldrich) supplemented with 10% fetal bovine serum (Gibco), 10 mM Hepes (Sigma–Aldrich), 1 mM sodium pyruvate (Sigma–Aldrich), and 0.1 mM nonessential amino acids (Sigma–Aldrich).

### Transient transfection of HEK293T cells with human *SLC39A8* genetic variants

Cell culture plates were coated with poly-d-lysine (0.1 mg/μl) (Sigma–Aldrich). Next, HEK293T cells were plated onto the coated 6-well (1,000,000 cells/well in 3 ml) or 96-well plates (55,000 cells/well in 100 μl). After 24 h, the cells were transfected with FuGENE HD (Promega) according to the manufacturer’s standard protocol (6-well plate: 3 μg DNA, 9 μl FuGENE HD, filled up to 155 μl with OptiMem [Sigma–Aldrich]/96-well plate: 0.1 μg DNA, 0.3 μl FuGENE HD, filled up to 10 μl with OptiMem). Experiments were performed 24 h after the transfection. Prior to the experiments, transfection efficiency was assessed by visual inspection of the expression of the dsRED fluorescent protein.

### Cell-surface biotinylation and Western blotting

Cells, seeded and transfected as described above for 6-well plates, were washed with PBS and incubated with 1.5 mg/ml (1 ml per well) sulfo-NHS-SS-biotin dissolved in biotin buffer (10 mM TEA [pH 7.4], 2 mM CaCl_2_) for 1 h at 4°C. Cells were then rinsed with PBS–Ca–Mg (PBS supplemented with 1 mM MgCl_2_, 0.1 mM CaCl_2_) and incubated with quenching buffer (PBS supplemented with 1 mM MgCl_2_, 0.1 mM CaCl_2_, 100 mM glycine) for 20 min. Cells were then lysed with radioimmunoprecipitation assay buffer (150 mM NaCl, 5 mM EDTA, 1% Triton X-100, 0.5% deoxycholate, 0.1% SDS, 50 mM Tris–HCl; pH 7.4 at room temperature) supplemented with cOmplete protease inhibitor cocktail (Roche Applied Science). Protein levels were determined, adjusted, and then incubated overnight at 4°C with an equivalent amount of streptavidin-coated agarose beads (100 μl beads in suspension [50:50] for 1 mg protein) dissolved in bead solution (50 mM Tris–HCl [pH 7.4], 100 mM NaCl, and 5 mM EDTA) (Thermo Fisher Scientific). The beads were washed several times and recovered by centrifugation with wash solutions A (1 mM Tris–HCl [pH 7.4], 5 M NaCl, 0.5 M EDTA), B (1 mM Tris–HCl, 5 M NaCl), and C (1 mM Tris–HCl). Biotinylated proteins were released by heat (95°C) in the presence of 2x Laemmli buffer.

Protein samples were loaded onto 12% SDS polyacrylamide gels, separated by electrophoresis, and transferred to a polyvinylidene fluoride membrane (Amersham). The membranes were then sequentially incubated with primary antibodies for ZIP8 (Merck Millipore; ABF 197, rabbit, 1:500 dilution) or ATPase chain A Na^+^/K^+^ (Merck Millipore; rabbit, 1:500 dilution) and the corresponding horseradish peroxidase–conjugated anti-rabbit secondary antibodies (Merck Millipore; mouse, 1:1000 dilution). Membranes were stripped by the high pH method (0.4 M NaOH) and incubated with HRP–avidin antibody (Bio-Rad) for biotin control. To assess expression levels of each of the transfected constructs, the resulting bands were quantified by densitometry using ImageJ (version 1.53q) (https://imagej.net/ij/).

### ^55^Fe-radioisotope Fe uptake

HEK293T cells were cultured in white, flat, and clear-bottom 96-well plates and transiently transfected as described above. First, the culture media were removed, and the cells were washed three times with ^55^Fe-uptake buffer (140 mM NaCl, 2.5 mM KCl, 1 mM CaCl2, 1 mM MgCl_2_, 1.2 mM K_2_HPO_4_, 10 mM glucose, 5 mM Hepes, 5 mM Mes; pH 7.4) at room temperature. ^55^Fe-uptake buffer supplemented with the indicated concentration of Fe, 1 mM ascorbic acid, and radioactively labeled Fe (final concentration of 0.5 μCi/ml) was added and incubated with the cells (100 μl/well) for 15 min at room temperature. To stop the reaction, the radioactive ^55^Fe-uptake buffer was removed, and the cells were washed three times with ice-cold ^55^Fe-uptake buffer. Finally, 100 μl of MicroScint-20 (PerkinElmer) was added to each well and incubated for another hour with constant shaking. The radioactive decay of ^55^Fe was measured using the MicroBeta2 plate counter (PerkinElmer) and analyzed as previously reported.^36^

### Intracellular Cd^2+^ accumulation recording by the FLIPR

HEK293T cells were cultured in black, flat, clear-bottom 96-well plates and transiently transfected as mentioned above. First, the culture media were removed, and the cells were washed three times with NCF buffer (117 mM NaCl, 4.8 mM KCl, 1 mM MgCl_2_, 10 mM glucose, 5 mM Hepes, and 5 mM Mes). Calcium-5 dye (Molecular Devices) was diluted in NCF buffer according to the manufacturer’s instructions. The diluted dye (50 μl) was added to each well and incubated at room temperature for 1 h. NCF buffer containing the indicated divalent metal ions and concentrations was prepared in a separate compound plate. Once the incubation time elapsed, the cell and compound plates were transferred to the FLIPR Tetra system (Molecular Devices), and changes in fluorescence intensity were recorded for 15 min. Binding of intracellular Cd^2+^ to the Calcium-5 dye results in a proportional fluorescent signal increase (excitation, 470–495 nm; emission, 515–575 nm). The transport activity was quantified as the area under the curve of the fluorescence signal induced by the accumulation of Cd^2+^ in the cells.

### Intracellular pH changes induced by HCO_3_^-^ uptake

Bicarbonate uptake was measured fluorometrically using the intracellularly trapped pH-sensitive dye BECEF (Thermo Fisher Scientific; B1170). HEK293T cells were cultured on glass coverslips in clear 6-well plates and transfected as described previously. The coverslips were washed three times with FluoroMax buffer A (115 mM NaCl, 5 mM KCl, 1.54 MgCl_2_, 1.1 mM CaCl_2_, 30 mM Hepes; pH 7.4 at room temperature). The cells were acidified with 20 mM NH_4_Cl in FluoroMax buffer A together with 1 μM BCECF for 30 min at 37 °C. The cells were then washed twice with FluoroMax buffer B (FMB) (115 mM choline chloride, 5 mM KCl, 1.54 MgCl_2_, 1.1 mM CaCl_2_, 30 mM Hepes; pH 7.4 at room temperature) and placed in a cuvette containing FMB buffer. After 150 s of background measurement, the FMB buffer was replaced with FluoroMax buffer C (115 mM NaCl, 5 mM KCl, 1.54 MgCl_2_, 1.1 mM CaCl_2_, 30 mM Hepes, 25 mM NaHCO_3_, pH 7.4 at 25 °C; pH 7.4 at room temperature). BECEF fluorescence (ƛ excitation = 490 nm and 440 nm, emission = 535 nm) was recorded for 7.5 min in the FluroMax2 spectrofluorometer (Photon Technology International). The rate of pH change over time was calculated from the slope of the curve. A calibration curve (pH between 4.5 and 8.0 in 0.5 increments) was used to calculate the actual pH values.

### Homology-based model of human ZIP8

A protein–protein BLAST search was performed to identify similar proteins with available crystallographic structures. The atomic resolution of BbZIP (PDB ID: 5TSA) (50) was used as a template for homology-based modeling. In a first step, the protein sequences were aligned (BLAST [version 1.2]—National Institutes of Health/Clustal Omega [version 1.2.4]—UCD/AlignMe [version 1.2]—National Institutes of Health/Modeller [version 9.12]—University of California/Jalview [version 2.11]—University of Oxford). The alignment was then verified using Jalview sequence alignment software and the protein sequence from the BbZIP crystal structure. The alignment of BbZIP and human ZIP8 was used for modeling (MODELLER [version 9.12]). In a first step, the TMHs of ZIP8 were superimposed on the 3D structure of BbZIP. Discrete optimized protein energy was used to evaluate the best protein model. In a second step, the large loop between TMH 3 and 4 was modeled. The final protein model was then evaluated again using the discrete optimized protein energy score. Secondary structure prediction servers (CFSSP [biogem.org], Jpred 4 [compbio.dundee.ac.uk], MESSA [prodata.swmed.edu], octopus [octopus.cbr.su.se], and predict protein [predictprotein.org]) were used to verify the position of the helices from ZIP8.

### Molecular docking of HCO_3_^-^ with ZIP8 homology model

The homology-based 3D model of human ZIP8 was used for molecular docking with AutoDock Vina 1.2 (AutoDock Vina [version 1.2]—Scripps Research Institute/AutoDock Tools [version 1.57]—Scripps Research Institute). AutoDock Vina uses a rectangular box to define the binding site. This is called the receptor region or search base. To define the box (10 × 10 × 8 Å), AutoDock Tools was used, and the predicted binding site of ZIP8 was selected. Because Zn^2+^ was the only transition metal ion optimized in Autodock Vina, only Zn^2+^ was used as the ligand metal ion in the docking process. In addition, His, Asp, and Glu were deprotonated and Arg, Lys were protonated. The prepared structure was then exported in PDBQT format, ready to use with Autodock Vina. After the binding site was defined, the ligand (HCO_3_^-^) was prepared according to Autodock Vina. The 3D structure file of HCO_3_^-^ was downloaded from PubChem in SDF format. This was converted to PDB format with the free conversion tool from www.cheminfo.org. In Autodock Tools, the ligand was prepared for the docking. The program automatically recognizes rotatable bonds inside the ligand (HCO_3_^-^). HCO_3_^-^ was then exported as well to PDBQT format, and the docking was performed. The results were evaluated with Autodock Tools. We searched especially for possible interactions (H-bonds) inside the estimated binding site.

### MD simulations of human ZIP8 with Zn^2+^ and HCO_3_^-^ bound to the proposed binding sites

The best-fitting results from the molecular dockings were used as the basis for the MD simulations. First, the orientation of the protein in the membrane was estimated using PPM2.0 (73). The systems for the simulations were created using CHARMM-GUI (2023; Lehigh University) (74, 75). A pure palmitoyl–oleoyl–phosphatidyl–choline rectangular membrane was chosen as the base. In addition, 150 mM NaCl was added. Finally, the CHARMM36m force field was selected for the simulations, and the temperature was set to 303.15 K. The simulations were run on the HPC cluster UBELIX at the University of Bern using the AMBER tools (Amber20, AmberTools22; https://ambermd.org/), with a total simulation length of 200 ns. The calculation steps were set to an interval of 2 fs. Simulations were run in chunks of 10 ns at a time, with a new run starting based on the previous calculation to avoid restarting from the beginning in case of crashes. These chunks were combined into a unified trajectory file using the AMBER20 module cpptraj.cuda. In the end, the trajectory files had a resolution of 10 frames per nanosecond. The resulting trajectories were further analyzed using the MD analysis Python module (76). First, the individual trajectories of the Zn^2+^ and HCO_3_^-^ ions were observed. A 5 Å zone was defined around the initial position of the ligands. If this zone was exceeded, it was considered as diffusion out of the binding site. Another boundary was drawn 15 Å. If this was also exceeded, the ligand was considered free and no longer bound within ZIP8. Next, the ions present within ZIP8 were counted, thus also determining the charge of the ions in the protein. In addition, the stability of the protein was monitored. This was done by measuring the RMSD value of the protein.

### Statistics

To assess the normal distribution of the data collections, Kolmogorov–Smirnov (N > 50) or Shapiro–Wilk (N < 50) statistical tests were applied. For normally distributed data, comparisons between two groups were made using an unpaired Student’s *t* test, and multiple-group comparisons were performed using one-way ANOVA followed by Tukey’s *post hoc* test. For nonparametric data, the Mann–Whitney *U* test was used for comparisons between two groups, and the Kruskal–Wallis test followed by Dunn’s *post hoc* test for multiple comparisons. Statistical analyses were performed using GraphPad Prism 8.0 (GraphPad Software, Inc).

## Data availability

All relevant data are included in this publication, but if for any reason additional information is desired, please contact Sven Baumann at svenp.baumann@gmail.com.

## Conflict of interest

The authors declare that they have no conflicts of interest with the contents of this article.
